# Chirality Transfer and Oxazolidine Formation in Reaction of L and D Enantiomers of β-Hydroxy Amino Acids with Nitrogenous Carboxaldehydes and Nickel(II)

**DOI:** 10.3390/molecules30142913

**Published:** 2025-07-10

**Authors:** Cynthia T. Brewer, Greg Brewer, Raymond J. Butcher

**Affiliations:** 1 Department of Chemistry, Catholic University, Washington, DC 20064, USA; brewerc@cua.edu; 2 Department of Chemistry, Howard University, Washington, DC 20059, USA; rbutcher99@yahoo.com

**Keywords:** chirality transfer, oxazolidine, threonine, beta hydroxy amino acids, homochirality

## Abstract

The reaction of either the L (*2S3R*) or D (*2R3S*) enantiomers of H_2_N-C*H(R)CO_2_^−^ (R = -C*H(OH)CH_3_ or -C*H(OH)CH(CH_3_)_2_) and the L (*2S*) or D (*2R*) enantiomers of H_2_N-C*H(C(CH3)_2_OH)CO_2_^−^ with imidazole-4-carboxaldehyde and nickel(II) acetate in methanol yields a single stereoisomer of an oxazolidine. There is retention of chirality on ring positions 4 and 5 (if C_β_ is chiral) of the oxazolidine, C_α_ and C_β_ of the parent amino acid, and transfer of chirality to the newly generated stereogenic centers, ring positions 3, the amino acid nitrogen atom, N_AA_, and 2, the aldehyde carbon atom, C_ald_. Specifically, when C_α_ has an *S* configuration, both N_AA_ and C_ald_ are formed as *R*. Likewise, a C_α_ which is *R* results in both N_AA_ and C_ald_ being formed as *S*. For example, the reaction of L threonine (C_α_ is *S* and C_β_ is *R*) with 4-imidazolecarboxaldehyde in the presence of nickel(II) gives the facial Λ NiL_2_, where L is (*2R*, *3R*, *4S*, *5R*) 4-carboxylato-5-methyl-2-(4-imidazolyl)-1,3-oxazolidine. The same reaction with D threonine produces the enantiomeric Δ complex of (*2S*, *3S*, *4R*, *5S*) 4-carboxylato-5-methyl-2-(4-imidazoyl)-1,3-oxazolidine. The high stereospecificity is thought to be based on the fused three-ring structure of the characterized nickel complexes in which the hydrogen atoms of C_α_, N_AA_, and C_ald_ must be cis to one another. Identical reactions occur with 2-pyridine carboxaldehyde and LT or DT. In contrast, the reactions of L allo threonine (*2S3S*) and the primary alcohols, L or D serine, give the conventional meridionally coordinated aldimine product.

## 1. Introduction

**Figure 1:**
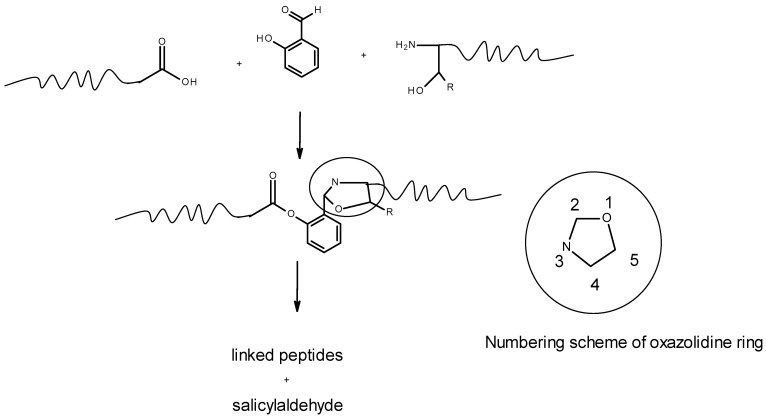
Sketch of serine (R = H) threonine (R = CH_3_) ligation (STL) method of coupling an N terminal residue with S or T residue to another polypeptide. The proposed mechanism goes through an oxazolidine, whose numbering is depicted.

The five-membered oxazolidine ring and its metal complexes have been recently investigated for asymmetric catalysis [1,2], antimicrobial effects [3], DNA binding [4], anticancer effectiveness [5,6] and a variety of industrial uses [7,8,9]. Oxazolidines are most commonly formed by reaction of a 1,2 amino alcohol, NH_2_CHRCHR’OH, with an aldehyde to give an imine which cyclizes on attack of the hydroxyl nucleophile on the electrophilic imine carbon atom [10,11]. Oxazolidine ring formation is also an integral step in serine (S) threonine (T) ligation (STL), which is a solid-state synthetic method for coupling two smaller polypeptides to form a larger polypeptide [12,13,14,15]. STL (see Figure 1) involves the formation of a salicylaldehyde ester on the carboxylic acid end of one polypeptide chain and subsequent imine formation by reaction of the aldehyde with the amino group of an incoming polypeptide having an N terminal S or T residue. Attack of the S or T hydroxyl group on the electrophilic imine carbon atom and proton transfer gives an oxazolidine ring. Acyl transfer and hydrolysis of the salicylaldimine fragment complete the coupling of the two original polypeptides.

Despite the success of STL, there are no reported oxazolidines in the Cambridge Structural Database (CSD) prepared from salicylaldehyde or derivatives with S or T. Schiff base condensates of S and T with salicylaldehyde form the aldimine, not an oxazolidine [16,17,18,19,20], and bind meridionally to a metal. For all the known S or T Schiff base complexes of an aldehyde, other than formaldehyde, the hydroxyl group of the beta carbon atom is not bound to the metal ion, and the -CH_2_OH or -CH(CH_3_)OH group simply branches off C_α_ and does not interact with the imine carbon or metal atom. The failure to isolate oxazolidines in the reaction of S or T with salicylaldehyde may be due to the poor electrophilicity of the aldehyde carbon atom [21,22]. In contrast to reactions of the poorly electrophilic salicylaldehyde, the very electrophilic formaldehyde reacts with both T [23] and metal complexes [24,25,26] of S and T to form oxazolidines.

**Figure 2:**
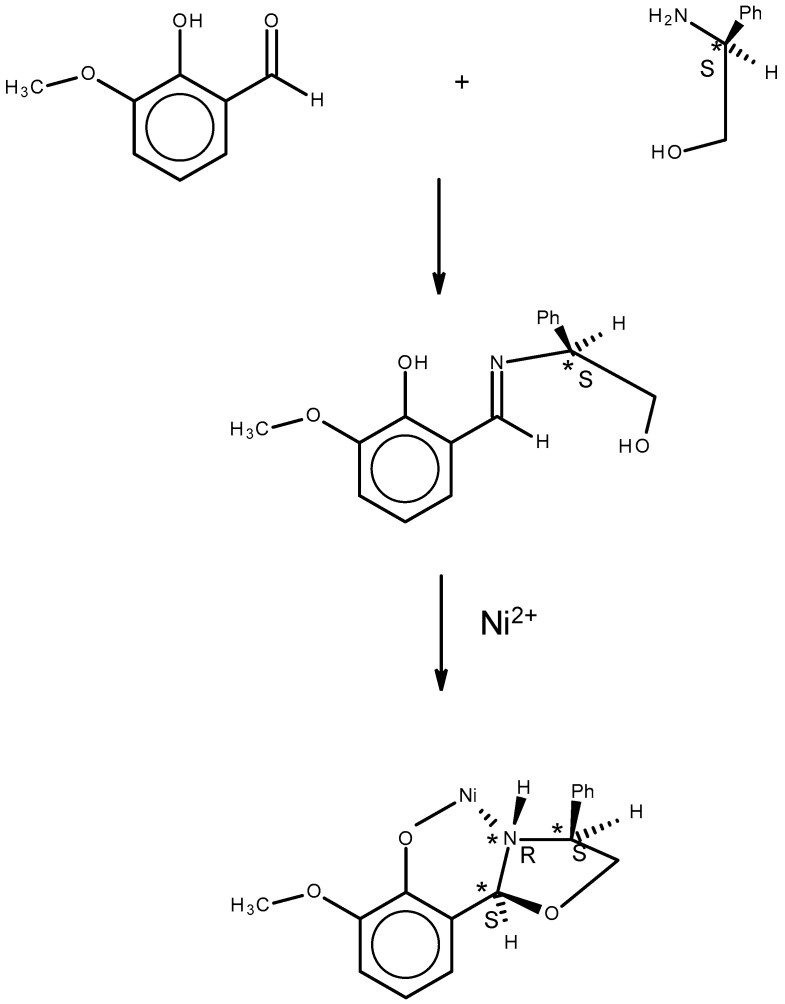
Chirality transfer in the reaction of chiral phenylglycinol with a salicylaldehyde, a prochiral aldehyde. On further reaction with nickel(II), the imine ligand gives the illustrated oxazolidine. Note the three-atom sequence in the product, C*_Phenyl glycinol_-N*_Phenylglycinol_-C*_ald_, is *SRS* or *RSR* for *S* or *R* phenylglycinol, respectively. An * after the symbol of an atom indicates that it is chiral.

A limitation of the product of the reaction of an amino alcohol or a beta hydroxy amino acid, βOHAA, such as T, with the very electrophilic formaldehyde is that the resultant oxazolidine would not contain a stereogenic center at ring position 2 (the former aldehyde carbon atom). Thus, chirality transfer [27,28,29], the creation of new stereogenic center(s) with a specific R or S configuration in an achiral substance on reaction with a chiral substance, cannot be observed with formaldehyde because it is not a prochiral aldehyde. A striking example of chirality transfer is observed in a reaction of a prochiral aldehyde [30], as described below and shown in Figure 2. The reaction of (*R* or *S*) phenyl- glycinol, NH_2_C*H(Ph)CH_2_OH, with a methoxy salicylaldehyde in the presence of nickel(II) resulted in oxazolidine complexes with three adjacent stereogenic centers, C*_Phenylglycinol_-N*_Phenylglycinol_-C*_ald_.

The chirality of the oxazolidine complexes was determined by the chirality of the commercially available enantiomerically pure *R* or *S* phenylglycinol starting material. *S* phenylglycinol gave only *SRS* for the C*_Phenylglycinol_-N*_Phenylglycinol_-C*_ald_ sequence, and the *R* starting material gave only the *RSR* product. It is not clear from the structures of these products why this stereochemical outcome occurs, but it does occur.

This work significantly extends the examples of chirality transfer through the preparation of an oxazolidine via the reaction of less reactive beta hydroxy amino acids, βOHAA (compared to 1,2 amino alcohols), with a sufficiently electrophilic [21,22] prochiral aldehyde, R-CH=O. βOHAAs are useful for several reasons. βOHAAs are readily available as both *R* and *S* enantiomers. Their use introduces the carboxylic acid functional group (or its derivatives) into the ring and a stereogenic center at ring position 4, C_α_. Ring position 5, C_β_ of the AA, may also be a stereogenic center, and the substituent on it can be varied. The parent prochiral aldehyde carbon atom (C_ald_) at ring position 2 introduces both another stereogenic center and another substituent, which can also be varied, at this position. Successful use of the βOHAA, as shown here, creates oxazolidines with multiple stereogenic centers (ring positions 2, 3, 4, and 5) and a variety of substituents at ring positions 2 (C_ald_), 4 (C_α_), and 5 (C_β_).

## 2. Results and Discussion

### 2.1. Approach, Ligands Employed, and Reactions Examined

**Figure 3:**
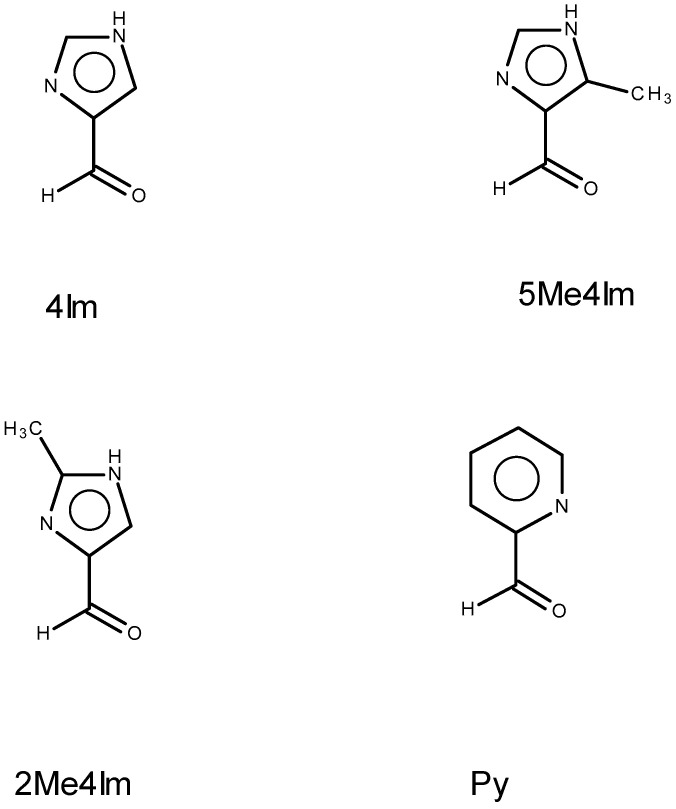
Line drawings and abbreviations of aldehydes employed in this study.

As mentioned in the introduction, the use of formaldehyde in reactions with βOHAA gives oxazolidines that lack a stereogenic center at ring position 2. Furthermore, while the use of salicylaldehyde would give a stereogenic center here, its reaction gives only imines and, to date, has not resulted in oxazolidines. The approach reported here to address the two problems of the relatively low electrophilicity [21,22] of salicylaldehyde with S or T to form oxazolidines and the lack of a prochiral atom at ring position 2 of the oxazolidine was to investigate the reaction of the nitrogenous carboxaldehydes shown in Figure 3. The use of these aldehydes addresses both issues. The inductive effect of the electronegative nitrogen atom in the aromatic ring may enhance the electrophilicity of C_ald_ to nucleophilic attack by the hydroxyl group of the β carbon atom. Additionally, their C_ald_ atoms are prochiral, which may allow for observation of chirality transfer.

**Figure 4:**
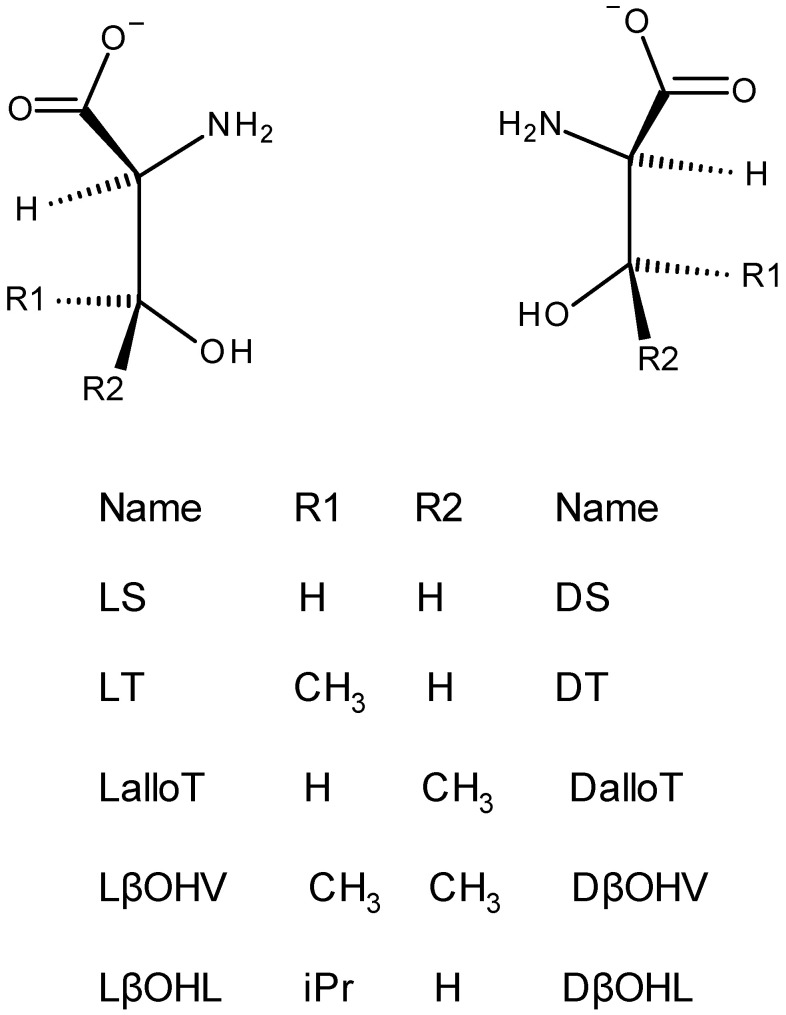
The L ((**left**) hand side) and D ((**right**) hand side) enantiomers of the five βOHAAs examined. One-letter symbols are used for serine (S), threonine (T), valine (V), and leucine (L). For LT and LβOHL, C_α_ is *S* and C_β_ is *R*, while for the D enantiomers, C_α_ is *R* and C_β_ is *S*. For L and D, βOHV C_α_ is *S* and *R*, respectively, and C_β_ is achiral. For LalloT, both C_α_ and C_β_ are *S*, and in the enantiomer, both are *R*.

The βOHAAs selected for reactions with the nitrogenous prochiral aldehydes are sketched in Figure 4. All are commercially available as the L and D enantiomers which provide a stereogenic center at ring position 4, C_α_. In addition, three of the βOHAAs have a stereogenic center at ring position 5, C_β_.

**Figure 5:**
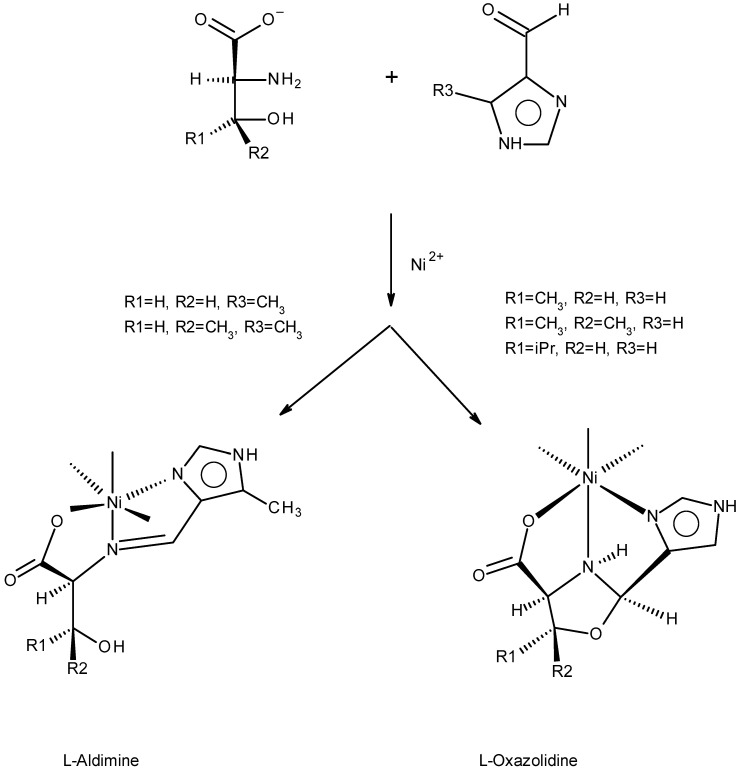
The reaction of the L enantiomer of the βOHAAs with either 4Im or 5Me4Im. The products of LS with 5Me4Im and LT with 4Im are labeled Ni(LS^Ald^5Me4Im)_2_ and Ni(LT^Ox^4Im)_2_, respectively. Use of the D enantiomer of the βOHAA results in formation of the enantiomer of the pictured product.

Reactions were conducted with a nitrogenous carboxaldehyde from Figure 3 and a βOHAA from Figure 4. Not every combination of a nitrogenous carboxaldehyde and a βOHAA was examined, in part due to cost of the βOHAAs, but also to concentrate on crystalline products from which chirality transfer information might be obtained. Most reactions were conducted with 4Im or 5Me4Im as these proved most suitable for forming crystals. In a reaction, equimolar amounts of the anions of the βOHAAs were refluxed with an aldehyde in aqueous methanol for thirty minutes followed by the addition of half an equivalent of nickel(II) acetate. All of the reaction mixtures were blue initially and over time deposited blue or purple crystals. These products were subsequently determined to be aldimine (blue) and oxazolidine (purple) as shown in Figure 5.

In the reaction of a βOHAA (an amine and also a 1,2 amino alcohol) with an aldehyde, there are two equilibria involved: the amine and aldehyde to give an aldimine (imine) and the amino alcohol and an aldehyde to give an oxazolidine. Figure 5 states which isomeric product, aldimine or oxazolidine, is formed for the reactions examined. Under different reaction conditions, other outcomes may be observed. No mechanistic studies are included in this report; only the characterization of the products is provided. The abbreviations of the ligands, aldimine or oxazolidine, are simply the symbols of the βOHAA (Figure 4) followed by that of the aldehyde (Figure 3). For example, the complexes prepared by reaction of L serine with 5Me4Im and L threonine with 4Im are Ni(LS^Ald^5Me4Im)_2_ and Ni(LT^Ox^4Im)_2_, respectively. The superscript of Ald or Ox after the βOHAA symbol serves as a reminder to the reader that the product is an aldimine or an oxazolidine.

The six L and D complexes of T, βOHV, and βOHL with 4Im and the two L and D complexes of T with Py presented here are unprecedented as they are the first report of oxazolidine formation from the reaction of a βOHAA with a prochiral aldehyde. Further, transfer of chirality is observed in all products, not only to ring position 2, the former C_ald_, but also to ring position 3, N_AA_.

### 2.2. Preliminary Characterization

FTIR spectra were recorded and provided in the ESI for every crystalline sample. These were not very helpful as all of the complexes show a strong carboxylate band and also all crystallize as hydrates which have broad absorption that overlap with N-H and C-H regions of interest. EA data and ESIMS were obtained for one of the two enantiomers of every βOHAA complex and provide independent determinations of the formulas. There was close agreement between the found and calculated values for the %C, %H, and %N values. ESIMS was useful, as a prominent molecular ion—[M + H]^+^, [M + Na]^+^, or [M + K]^+^—was always observed. UV-vis spectra were not obtained as the solids obtained from the reaction mixtures were not sufficiently soluble to obtain spectra. This insolubility also precludes the acquisition of NMR, CD, or optical rotation data.

### 2.3. Nickel Coordination Environment

**Figure 6:**
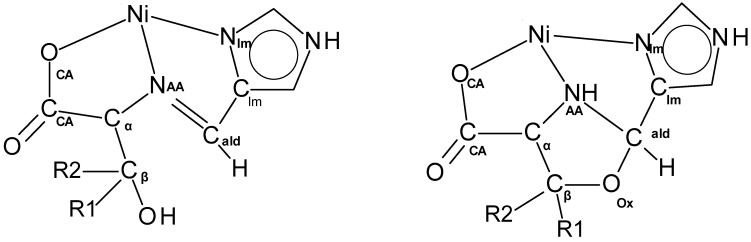
Labeling of the donor atoms of the aldimine (**left**) and oxazolidine (**right**) complexes, O_CA_, N_AA_, and N_Im_ and the other ligand atoms that define the chelate rings, C_CA_, C_α_, C_β_, O_Ox_, C_Im_, and C_ald_. If Py rather than Im is used as the aldehyde, the N_Im_ and C_Im_ symbols are replaced with N_Py_ and C_Py_.

The crystallographic information for the eight oxazolidine and three aldimine complexes is given in Table S1 in the ESI. In all of the eleven reported complexes, the nickel(II) ion is coordinated by two identical N_2_O ligands. The NiN_4_O_2_ coordination environment is a slightly distorted octahedron. Selected structural parameters to illustrate the coordination geometry are given in Table S2. The donor atoms, N_Im_ (or N_Py_), N_AA_, and O_CA,_ as well as the other atoms discussed, are shown in Figure 6.

Structural similarities and differences between the aldimine and oxazolidine complexes are given below. There is no significant structural difference among the structures of the present aldimine complexes, Ni(LS^Ald^5Me4Im)_2_, Ni(DS^Ald^5Me4Im)_2_, and Ni(LalloT^Ald^5Me4Im)_2_, and those reported earlier from reactions of an AA and 2-pyridinecarboxaldehyde [31] or 4-imidazole carboxaldehyde [32,33]. All are meridionally coordinated through the O_CA_, N_AA_, and N_Im_ (or N_Py_) atoms with a trans N_AA_-Ni-N_AA_′ angle > 170° and trans O_CA_-Ni-N_(Py/Im)_ of ~156°. Meridional, rather than facial, coordination is required due to the rigid backbone of the ligand imposed by the imine double bond in the middle of the ligand between N_AA_ and C_ald_. No further discussion of the three new blue aldimine complexes is provided as they do not represent a new structural motif. A discussion of reactivity differences between the three βOHAAs that give an aldimine (LS and DS and LalloT) and the six (L and D) complexes of T, βOHV, and βOHL that give an oxazolidine is provided in Section 2.6. There are no previously reported meridionally coordinated complexes of a βOHAA with either a pyridine or an imidazole carboxaldehyde. However, with a low-valent metal, the Schiff base complexes of 2-pyridinecarboxaldehyde with serine are bound in a bidentate manner through N_Py_ and N_AA_ [34].

**Figure 7:**
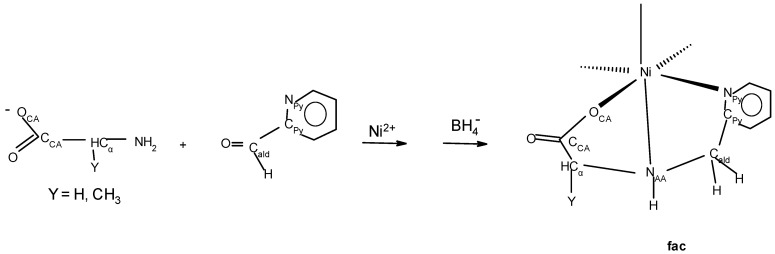
The reaction of the anion of glycine or L alanine with 2-pyridinecarboxaldehyde gives the aldimine complex which on addition of Ni(II) and reduction of the imine gives facial coordination of the ligand.

In the eight unprecedented oxazolidine complexes, the nickel(II) ion is bound in a cis facial (cis N_AA_ and N_AA_′ atoms) manner [35] with trans axes of N_Im/Py_-Ni-N_Im/Py_, O_CA_-Ni-N_AA_′, and O_CA_′-Ni-N_AA_. In order for the Schiff base of an AA and a carboxaldehyde to coordinate in a facial manner, the planar aldimine ligand must fold together along its M-N_AA_ axis so that the O_CA_-Ni-N_Im/Py_ angle of the ligand decreases from ~156° to ~90°, which requires breaking of the N_AA_=C_ald_ double bond. Facial coordination has been observed in the nickel complexes of PyCH_2_NHCH(R)CO_2_^−^ (R = H or CH_3_) [36] (O_CA_-Ni-N_Py_ of ~90°) prepared by borohydride reduction of PyCH=NCH(R)CO_2_^−^, as shown in Figure 7.

In the present oxazolidine complexes, the N_AA_=C_ald_ double bond is replaced by the N_AA_-C_ald_ and O_ox_-C_ald_ single bonds through formation of the oxazolidine ring. Both PyCH_2_NHCH(Y)CO_2_^−^ and the present oxazolidine complexes have (N_Im/py_N_AA_O_CA_)_2_ donor sets, are purple, exhibit *cis* facial coordination with *trans* N_Im/Py_ atoms, and have~perpendicular N_Im/py_-Ni-O_CA_ angles. The difference between them is that in the present complexes, there is a two-atom linkage, C_β_-O_ox_, that connects C_α_ to C_ald_ as a result of oxazolidine formation, which drastically affects the overall shape.

### 2.4. Overall Shape of the Oxazolidine Complexes

**Figure 8:**
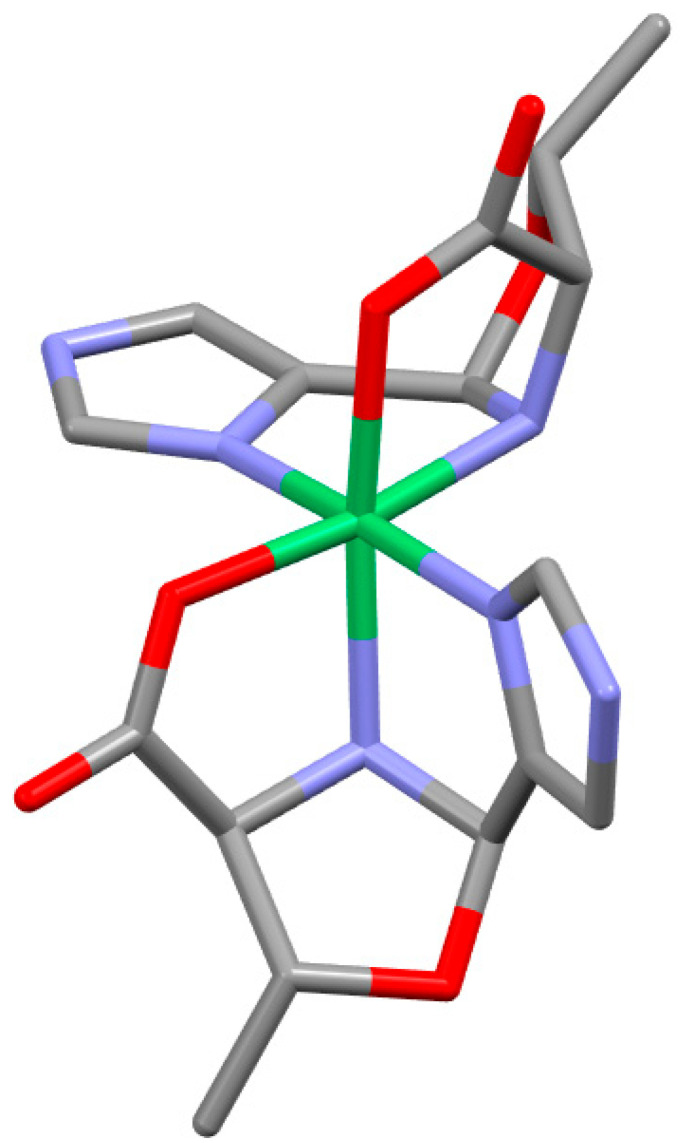
Ni(LT^Ox^4Im)_2_ with omission of hydrogen atoms for clarity. The pseudo-three-sided box is easily seen for the bottom ligand. The five-atom carboxylate and imidazole sides are on the left and right, respectively, with the oxazolidine ring on the bottom. Note that the N_AA_ atoms are *cis* and N_Im_ atoms are *trans*. Color codes: Ni (green); O (red); N (purple); C (gray).

**Figure 9:**
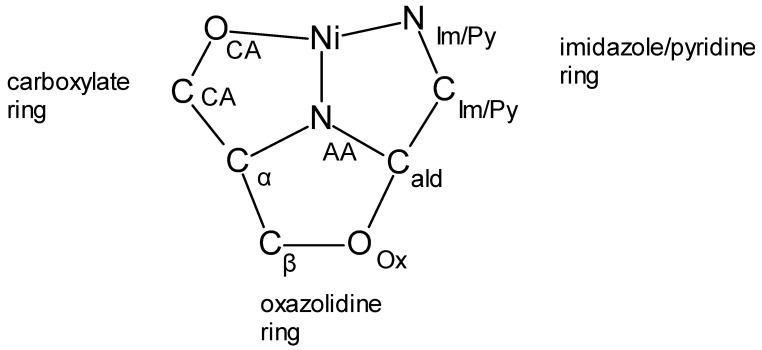
The labeling of the three fused five-membered rings and their component atoms present in all eight βOHAA complexes (L and D enantiomers). The rings are far from coplanar. The carboxylate and imidazole/pyridine rings are ~perpendicular to one another and form the left and right sides of the pseudo-three-sided box. The oxazolidine ring forms the bottom of the box and is bent down from the two sides.

Each of the eight tridentate oxazolidine ligands binds in a cis facial manner, resulting in the same shape for all of the complexes, as illustrated in Figure 8 for Ni(LT^Ox^4Im)_2_. Each of the two facially coordinated ligands creates a pseudo-three-sided box. The bottom ligand in the figure is oriented to provide a clear view of the three sides, each consisting of a five-atom ring. These rings and their component atoms are the carboxylate (Ni, O_CA_, C_CA_, C_α_, and N_AA_), imidazole (or Py) (Ni, N_Im/Py_, C_Im/Py_, C_ald_, and N_AA_), and oxazolidine (O_Ox_, C_ald_, N_AA,_ C_α_, and C_β_) rings. The carboxylate and imidazole (or Py) sides share an edge, Ni-N_AA_, and both share edges with the oxazolidine ring, C_α_-N_AA_ and N_AA_-C_ald_, respectively. The N_AA_ position is the only atom shared among the three pentagonal rings defined above. The labelings of the three rings and of all of the atoms in these rings are given in Figure 9.

**Figure 10:**
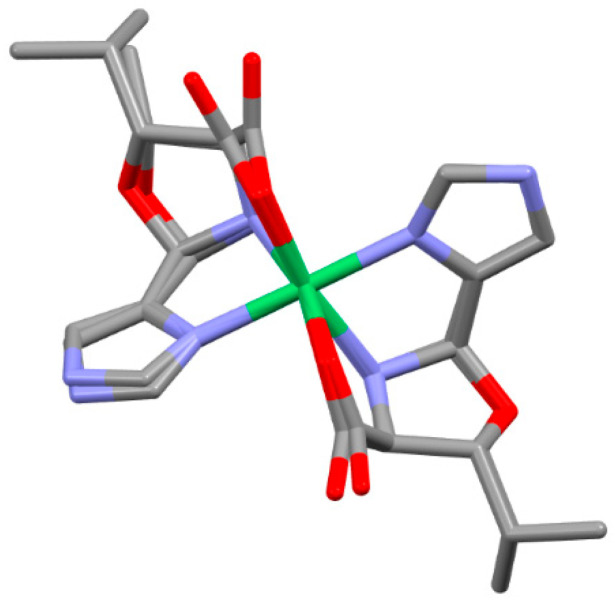
The overlayed structures of Ni(LT^Ox^4Im)_2_ and Ni(LβOHL^Ox^4Im)_2_. Hydrogen atoms have been omitted for clarity. The overlayed atoms are nickel and each of the six donor atoms in each complex. The RMS is 0.0861. Color codes: Ni (green); O (red); N (purple); C (gray).

The carboxylate and imidazole (or py) sides are each nearly planar, as indicated by the relatively small out-of-plane displacements of their atoms. They are also nearly perpendicular to one another, which is suggested by the average O_CA_-Ni-N_ImPy_ angles (Table S2) of 91.9°. The bottom of the three-sided box is the non-planar oxazolidine ring, which is bent down from the carboxylate and imidazole (or Py) sides presumably to reduce the crowding/strain resulting from formation of the three-sided box. All of the bond distances and angles for each of the carboxylate, imidazole/pyridine, and oxazolidine sides are given in Table S3 of the ESI for the four LβOHAA complexes. There is remarkably little variation in any of these structural parameters among these homologous complexes (and of course their enantiomers) which may be attributable to the restrictions imposed by three fused non-planar rings. This is illustrated by examining the overlayed structures of Ni(LT^Ox^4Im)_2_ and Ni(LβOHL^Ox^4Im)_2_ shown in Figure 10. As can be seen, there are only small differences between the positions of most atoms, which may be imposed by the three fused rings.

While this structural motif has not been reported for the βOHAAs until now, it has been observed in the product of the reaction of the very nucleophilic cysteine with 2-pyridinecarboxaldehyde [37].

### 2.5. Chirality Transfer

**Figure 11:**
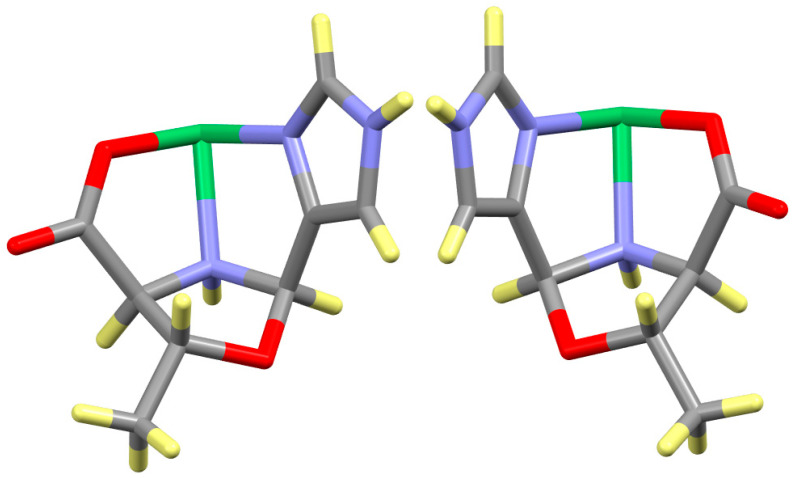
Diagram of Ni(LT^Ox^4Im)_2_ (**left**) and Ni(DT^Ox^4Im)_2_ (**right**) complexes. The two are mirror images of one another. Only one of the ligands is shown for clarity. The important C_α_N_AA_C_ald_ linkage is beneath the nickel and is *SRR* for LT and *RSS* for DT. C_β_ is in the foreground and is *R* and *S* for LT and DT, respectively. Color codes: Ni (green); O (red); N (purple); C (gray); H (yellow).

The most important aspect of these structures is the stereochemistry of the two newly generated stereogenic centers, N_AA_ and C_ald_, which is dictated by the constraints of the facial coordination of the ligand and the formation of the oxazolidine ring. This is most easily explained by examining the facial binding of one ligand of the Ni(LT^Ox^4Im)_2_ and Ni(DT^Ox^4Im)_2_ complexes, as depicted in Figure 11. The N_AA_ and C_ald_ atoms become stereogenic centers as each expands its coordination number from three to four on formation of the oxazolidine ring. The mechanism of formation of the oxazolidine ring has not been investigated for these reactions, but several changes must occur regardless of the sequence of reactions. C_ald_ becomes four-coordinate when it forms bonds with both N_AA_ and the hydroxyl group attached to C_β_ and it undergoes water loss. N_AA_ undergoes coordination expansion by picking up a hydrogen atom. In principle, either of the newly generated four-coordinate stereogenic centers, N_AA_ and C_ald_ (two on each ligand), could be either *R* or *S*. However, the constraints imposed by the facial coordination of the ligand and the formation of the oxazolidine ring require that N_AA_ and C_ald_ have the opposite stereochemical designation as C_α_, which is explained below.

The carboxylate (Ni-O_CA_-C_CA_-C_α_-N_AA)_ and imidazole/pyridine (Ni-N_Im/Py_-C_Im/Py_-C_ald_-N_AA_) rings are tethered to one another by the C_β_–O_OX_ linkage. These constraints require that the C_α_, N_AA_, and C_ald_ hydrogen atoms be *cis* to one another, giving an *SRR* assignment for the C_α_-N_AA_-C_ald_ sequence of the LT product. It is not possible for either of the N_AA_ or C_ald_ hydrogen atoms to be *trans* to the C_α_ hydrogen atom of LT, as this would require the inversion of both the N_AA_ and C_ald_ atoms, which are bonded together and also bound to other atoms in the carboxylate, pyridine, and oxazolidine sides. The only outcome for the eventual bond formation between the beta hydroxyl group and C_ald_ is to have the hydrogen atoms of N_AA_ and C_ald_ be *cis* to the C_α_ hydrogen atom. For the same reason, the C_α_, N_AA_ and C_ald_ hydrogen atoms of the DT product must also be *cis* to one another, giving an *RSS* assignment for the C_α_-N_AA_-C_ald_ sequence. For the oxazolidine produced from both LT or DT, the hydrogen atom of C_β_ is *trans* to the C_α_, N_AA_, and C_ald_ hydrogen atoms and allows the methyl group to be in the more open (in this case) *cis* position. The position of the methyl group in the LT or DT oxazolidine complexes is always cis to the C_α_ hydrogen atom and trans to the C_β_ hydrogen atom as required by the stereochemistry.

**Figure 12:**
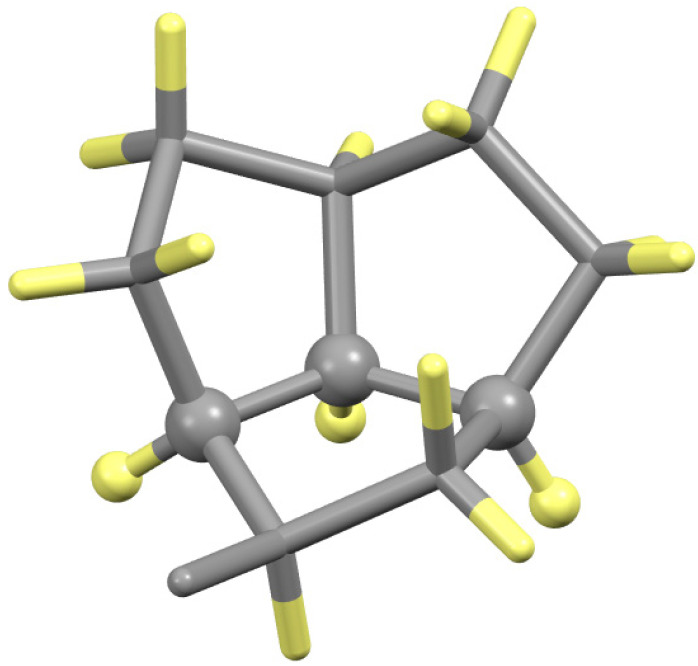
Structure of bivalvane, (C_10_H_15_)_2_, showing one of the two fused three five-membered rings. The three atoms that link the rings together and their hydrogen atoms are shown as spheres to highlight the linkage. Note that the three hydrogen atoms of the linkage are all *cis*, as are the present oxazolidine complexes. Color codes: C (gray); H (yellow).

This same result is found for the Ni(LT^Ox^Py)_2_ and Ni(DT^Ox^Py)_2_, Ni(LβOHV^Ox^4Im)_2_ and Ni(DβOHV^Ox^4Im)_2_, and Ni(LβOHL^Ox^4Im)_2_ and Ni(DβOHL^Ox^4Im)_2_ pairs of enantiomers as the same argument applies. The complexes of βOHV^Ox^4Im lack a stereogenic center at C_β_, but the assignment of N_AA_ and C_ald_ is still the opposite of that of C_α_ as observed for the other βOHAA^Ox^4Im complexes. The observation that the hydrogen atoms of C_α_, N_AA_, and C_ald_ are all *cis*, giving *SRR* and *RSS* for the three-atom sequence, of the LβOHAA^ox^ and DβOHAA^Ox^ complexes, respectively, is similar to what is found in the structure of the hydrocarbon, bivalvane [38], a proposed precursor to dodecahedrane, as shown in Figure 12. The present oxazolidine complexes and bivalvane are quite distinct but share an identical feature. They exhibit a pseudo-three-sided box in which each side is made up of five atoms, and the hydrogen atoms of the three-atom sequence that links the sides together (all C for bivalvane and C_α_, N_AA_, and C_ald_ for the oxazolidines) are *cis*.

Table 1 provides a summary of the remarkable stereospecificity observed for these complexes. LalloT (both C_α_ and C_β_ are *S*), which gives an aldimine, is a special case as explained in Section 2.6. Table 1 also shows that the Ʌ and Δ oxazolidine complexes correlate with a C_α_ of *S* and *R*, respectively. This type of transfer of chirality, also referred to as induced stereoselectivity, has been observed in complexes of amino acids [39,40,41] and is easy to understand. When multiple polydentate chiral L AA ligands chelate to a metal, the resulting metal complexes will be both Δ and Ʌ, while the configuration of C_α_ is locked in as *S* since the rate of racemization of C_α_ atoms is relatively slow. These metal chelates, designated as ΔC_α_(*S*) and ɅC_α_(*S*), are diastereomers, and unlike enantiomers, differ in energy and solubility. During crystallization, the less soluble form, either ΔC_α_(*S*) or ɅC_α_(*S*), is isolated since the rates of racemization for many metal chelates are fast. The preference for either ΔC_α_(*S*) or ɅC_α_(*S*) is a correlation of chirality based on either an energetic or solubility difference between the ΔC_α_(*S*) and ɅC_α_(*S*) diastereomers. For these oxazolidine complexes, ɅC_α_(*S*) is observed while ΔC_α_(*S*) is not. For the aldimine complexes, Table 1 shows a correlation of Δ with C_α_(*S*) and Ʌ with C_α_(*R*), which is opposite to that of the oxazolidines. This is not surprising as the aldimines and oxazolidines are different classes of compounds and cannot be compared directly. The observed chirality correlation of these aldimine complexes is identical to that reported earlier [32,33] for other aldimines of AA and imidazole aldehydes as anticipated.

### 2.6. Preliminary Reactivity Comparison

Based on the reported reactions, it is possible to perform a preliminary ordering of the reactivity of both the βOHAAs and the aldehydes that they condense with, which of course will need to be modified as additional data is gathered. Oxazolidine formation depends on the nucleophilicity of the C_β_-OH and the electrophilicity of C_ald_ of the nitrogenous carboxaldehyde. In addition to these considerations, reactivity can also be influenced by steric and other effects, which may be significant.

If the reactivity ranking of the βOHAAs was due entirely to their class (1°, 2°, or 3° alcohol), then the predicted ranking would be

βOHV (3° alcohol) > βOHL~T~alloT (2° alcohols) > S (1° alcohol)

as βOHV is a tertiary alcohol, βOHL, T, and alloT are secondary alcohols, and S is a primary alcohol. The additional alkyl groups on C_β_ are electron-releasing and should enhance the nucleophilicity of the C_β_-OH. However, the ordering based on reaction products and observed color changes in the present reactions is different from the above.

βOHV > βOHL~T >> S~alloT

βOHV, βOHL, and T all produce oxazolidine (greater reactivity), while S and LalloT produce aldimine (lower reactivity). Reaction mixtures of βOHV after several hours changed from blue(aldimine) to purple(oxazolidine) prior to depositing purple crystals. This suggests that oxazolidine formation occurred earlier in the reaction timeline relative to T and βOHL, which deposited purple crystals from a blue solution, suggesting that the conversion to oxazolidine occurred late in the reaction timeline and possibly during crystallization.

LalloT (*2S3S*) is a special case in that it is a secondary alcohol like T but gave an aldimine product as did S, a primary alcohol. Had LalloT reacted to give an oxazolidine, it would have resulted in placing a methyl group trans to the hydrogen atoms of C_α_, N_AA_, and C_ald_, that is “inside the box”. This could result in some unfavorable energetic steric interactions, which may shift the equilibria to the aldimine product. βOHV, which is the most reactive βOHAA, has two methyl groups on C_β_, so one of its methyl groups is trans to the hydrogen atoms of C_α_, N_AA_, and C_ald_ in the oxazolidine product. If this produces some steric energetic problem, as suggested for LalloT, why does it form the oxazolidine and why does LalloT form the aldimine? The greater reactivity of βOHV (tertiary alcohol) must overcome whatever steric interactions hinder alloT from forming the oxazolidine. It is important to consider that the AA and aldehyde reactants are in equilibrium with both the aldimine and the oxazolidine products. The isolated products reflect the equilibrium between aldimine and oxazolidine under the conditions employed. A reaction of LS with 5Me4Im and nickel(II) under several hours reflux still yielded only the aldimine as determined from its cell parameters. However, it may be possible to generate an oxazolidine from S or LalloT and a prochiral aldehyde under different, more vigorous, reaction conditions. The relative nucleophilicity of S and alloT cannot be ranked in terms of these reactions as clearly there is a steric concern (tethering of the side chain of alloT) that hinders its reactivity as discussed above.

The ordering of the aldehydes in terms of the electrophilicity of C_ald_ is

H_2_CO >> 4Im~Py~5Me4Im > 2Me4Im >> salicylaldehyde

The rankings of H_2_CO and salicylaldehyde are based on earlier studies where H_2_CO [23] reacts with S to form oxazolidine, and salicylaldehyde gives only aldimine when reacted with S or T [16,17,18,19,20]. No significant difference in reactivity was noted for reactions of 4Im, 5Me4Im, and Py with LT or DT based on color. The reaction mixtures were blue (aldimine) but deposited purple crystals (oxazolidine). The products with 4Im and Py were crystalline, and structural characterization revealed oxazolidine. The products of T with 5Me4Im were purple non-crystalline solids. Oxazolidine formation is not certain in this case, but the solid was purple as were other oxazolidines. More work is needed on these non-crystalline species.

A few reactions of 2Me4Im with LT, LalloT, and βOHV were also examined simply to extend reactivity comparisons to this aldehyde. The four structures of 2Me4Im complexes are given in the Supplemental Information as supplemental structures, as shown in Table S4. More work will be conducted on 2Me4Im as well as other aldehydes to expand the reactivity series of the aldehydes. The products from LT and LalloT were Ni(LT^Ald^2Me4Im)_2_ and Ni(LalloT^Ald^2Me4Im)_2_, respectively. The observation that LT gives an aldimine is surprising and supports the lower reactivity ranking of 2Me4Im relative to 4Im. The reaction of βOHV gave two products: Ni(LβOHV^Ald^2Me4Im)_2_ and Ni(LβOHV ^Ox^2Me4Im)(LβOHV). The former is also surprising as it is an aldimine produced from the most reactive LβOHV. The second product has two facially coordinated ligands: an oxazolidine and a free LβOHV. This is the first report of an underivatized facially coordinated tridentate βOHAA, which may be a key species in the conversion of a βOHAA in the presence of an aldehyde to an oxazolidine. There are structural reports of complexes of N alky derivatives [42,43] or an imine of a βOHAA [44].

## 3. Experimental Procedure

### 3.1. General

D and L Threonine, D and L serine, 4-imidazole carboxaldehyde, 2-pyridine carboxaldehyde, 4-methyl-5-imidazole carboxaldehyde, nickel(II) acetate tetrahydrate, methanol, and 0.10 M potassium hydroxide in methanol were obtained from Aldrich (St. Louis, MO, USA). (2R, 3S)-2-amino-3-hydroxy-4-methylpentanoic acid (β-hydroxy D-leucine) and L-allothreonine were obtained from Combi-Blocks (San Diego, CA, USA). (2S, 3R) 2-amino-3-hydroxy-4-methylpentanoic acid (β-hydroxy L-leucine) was obtained from Santa Cruz Biotechnology (Santa Cruz, CA, USA). (S) 2-Amino-3-hydroxy-3-methylbutanoic acid (β-hydroxy L-valine) was obtained from Advanced Chem Block (Hayward, CA, USA). All solvents were of reagent grade and used without further purification. Pyridine-2-carboxaldehyde was distilled prior to use. IR spectra were obtained using a Perkin Elmer Spectrum Two FT IR spectrophotometer.

### 3.2. External Laboratories

ESI-MS spectra were obtained by the Axis Pharm Laboratory (San Diego, CA, USA). EA data were obtained by Galbraith Laboratories, Inc. (Knoxville, TN, USA).

### 3.3. X-Ray Crystallography

Crystal data for all complexes were collected on a Rigaku Synergy-S single-crystal X-ray diffractometer. All structures were solved using the direct methods program SHELXS-97 [45]. All nonsolvent heavy atoms were located using subsequent difference Fourier syntheses. The structures were refined against F^2^ with the program SHELXL [46,47], in which all data collected were used including negative intensities. All nonsolvent heavy atoms were refined anisotropically. All hydrogen atoms were located by Fourier difference. Complete crystallographic details are given in Tables S1 and S4.

### 3.4. Synthesis

Ni(LT^Ox^4Im)_2_. To a solution of 5.0 mL of H_2_O, 10.0 mL of 0.100 M KOH in methanol (1.00 mmol), and 15.0 mL of methanol was added 0.119 g (1.00 mmol) of LT and 0.096 g (1.0 mmol) of 4-imidazole carboxaldehyde. This mixture was refluxed for 30–40 min, and then 10.0 mL of a 0.0500 M solution of nickel(II) acetate in methanol (0.500 mmol) was added. The reaction mixture turned blue and was set aside to concentrate. After several days, the reaction mixture was filtered to give purple crystals of the oxazolidine complex (yield = 0.114 g, 51%). All other reactions were conducted as described for Ni(LT^Ox^4Im)_2_. Table S5 contains the yield, elemental analysis data, and electron spray ionization data for a single enantiomer of each of the complexes reported in this study.

## 4. Conclusions

The reactions of LS, DS, and LalloT with 5-methyl-4-imidazolecarboxaldehyde, 5Me4Im, in the presence of nickel(II) give the unremarkable aldimine complexes, Ni(L or DS^Ald^5Me4Im)_2_ and Ni(LalloT^Ald^5Me4Im)_2_. Their failure to give the oxazolidine complexes is attributed to the poor nucleophilicity of S, a primary alcohol, and possible unfavorable steric interactions with LalloT. In stark contrast, the reactions of L and D threonine, L and D βOHV, and L and D βOHL with 4-imidazolecarboxaldehyde, 4Im, in the presence of nickel(II) give oxazolidines, Ni(L or D βOHAA^Ox^4Im)_2_. In addition, LT and DT give analogous complexes with 2-pyridinecarboxaldehyde, Ni(L or DT^Ox^Py)_2_. These eight complexes, four pairs of enantiomers, are unprecedented in that they are the only cases of the reaction of a βOHAA with a prochiral aldehyde to give an oxazolidine. In these cases, chirality transfer is observed. New chiral centers are generated at oxazolidine ring positions 2 (the former aldehyde carbon atom C_ald_) and 3 (the former amino acid nitrogen atom N_AA_). In all cases, the stereochemistry of the C_α_ and C_β_ (if chiral) atoms is conserved, and new stereogenic centers are created at the C_ald_ and N_AA_ atoms. Their assignments are opposite to that of the C_α_ atom so that the hydrogen atoms of C_α_, N_AA_, and C_ald_ are *cis* to one another and *trans* to that of C_β_. The transfer of chirality in these cases is thought to be linked to the fused three-ring structure which is quite rigid. It does not seem possible for the hydrogen atoms of C_α_ to be trans to either the N_AA_ or C_ald_ hydrogen atoms, given the rigidity of the non-planar three fused rings.

Each NiL_2_ complex contains two equivalent ligands and nine total stereogenic centers (with a chiral C_β_ atom), 256 pairs of enantiomers. These centers are C_α_ and C_β_, which are conserved from the reactant, and new centers, N_AA_ and C_ald_, which are determined by C_α_ (*S* gives *R* and the reverse) for each ligand and the nickel complex itself. Ʌ and Δ nickel complexes are produced from L and D βOHAA, respectively.

## Figures and Tables

**Table 1:** Assignments for stereogenic centers in complexes.

Compound	Complex	C_α_	C_β_	N_AA_	C_Ald_
Ni(LT^Ox^4Im)_2_	Ʌ	*S*	*R*	*R*	*R*
Ni(DT^Ox^4Im)_2_	Δ	*R*	*S*	*S*	*S*
Ni(LT^Ox^Py)_2_	Ʌ	*S*	*R*	*R*	*R*
Ni(DT^Ox^Py)_2_	Δ	*R*	*S*	*S*	*S*
Ni(LβOHV^Ox^4Im)_2_	*Ʌ*	*S*	*---*	*R*	*R*
Ni(DβOHV^Ox^4Im)_2_	Δ	*R*	*---*	*S*	*S*
Ni(LβOHL^Ox^4Im)_2_	Λ	*S*	*R*	*R*	*R*
Ni(DβOHL^Ox^4Im)_2_	Δ	*R*	*S*	*S*	*S*
Ni(LS^Ald^5Me4Im)_2_	Δ	*S*	*---*	*---*	*---*
Ni(DS^Ald^ 5Me4m)_2_	Λ	*R*	*---*	*---*	*---*
Ni(LalloT^Ald^5Me4Im)_2_	Δ	*S*	*S*	*---*	*---*

## Data Availability

All of the cif files for the reported complexes are deposited with the CSD of the CCDC, and the deposition numbers of all complexes are given in Supplemental Tables S1 and S4. The author will respond to any reasonable request to supply any additional information.

## Supplementary Materials

The following supporting information can be downloaded at https://www.mdpi.com/article/10.3390/molecules30142913/s1. The ESI contains tables for crystallographic data; bond distances and angles for the coordination sphere; bond distances and angles for the carboxylate, imidazole/pyridine, and oxazolidine rings; ESIMS and CHN analysis data; FTIR spectra of all complexes; and ESIMS spectra. Table S1: Crystallographic Data for eleven Nickel complexes of β hydroxy AA condensed with nitrogenous aldehydes; Table S2: Selected bond distances (Å) and angles (^o^) for the Ni(LβOHAA^Ox^4Im)_2_ and Ni (LT^Ox^Py)_2_ complexes; Table S3: Selected bond distances(Å) and angles (^o^) for the three five membered rings of the four Ni(LβOHAA^Ox^Py/4Im)_2_ complexes; Table S4: Crystallographic data for supplemental structures of 2Me4Im condensed with select beta hydroxy amino acids; Table S5: Summary of %yield, EA and ESI MS values for one enantiomer of the prepared complexes.
