# Supplementary material for: Chirality Transfer and Oxazolidine Formation in Reaction of L and D Enantiomers of β-Hydroxy Amino Acids with Nitrogenous Carboxaldehydes and Nickel(II)
Source: Molecules. 2025 Jul 10;30(14):2913. doi: 10.3390/molecules30142913 (PMC12298519; doi:10.3390/molecules30142913)
Supplement: Supplementary file 1 [file molecules-30-02913-s001.zip › molecules-3729019-supplementary.pdf]

Electronic Supplemental Information for

Chirality Transfer and Oxazolidine Formation in Reaction of L and D enantiomers of β-Hydroxy Amino Acids with Nitrogenous Carboxaldehydes and Nickel(II).

Cynthia T. Brewer[a], Greg Brewer[a*] and Raymond J. Butcher[b]

[a] Department of Chemistry, Catholic University, Washington DC USA

[b] Department of Chemistry, Howard University, Washington DC USA

Supplemental Table 1 Crystallographic Data for eleven Nickel complexes of β hydroxy AA condensed with nitrogenous aldehydes

Supplemental Table 2. Selected bond distances (Å) and angles (°) for the Ni(LβOHAA^{Ox}4Im)$_2$ and Ni(LT^{Ox}Py)$_2$ complexes.

Supplemental Table 3. Selected bond distances(Å) and angles(°) for the three five membered rings of the four Ni(LβOHAA^{Ox}Py/4Im)$_2$ complexes.

Supplemental Table 4. Crystallographic data for **supplemental structures of 2Me4Im** condensed with select beta hydroxy amino acids

Supplemental Table 5 Summary of %yield, EA and ESI MS values for one enantiomer of the prepared complexes

ESI MS for a single enantiomer of complexes

FTIR for β hydroxy AA condensed with 4Im, 5Me4Im and Py carboxaldehydes

Supplemental Table 1 Crystallographic Data for Nickel complexes of β hydroxy AA condensed with nitrogenous aldehydes

Compound	**Ni(LT^Ox^4Im)₂**	**Ni(DT^Ox^4Im)₂**	**Ni(LT^Ox^Py)₂**	**Ni(DT^Ox^Py)₂**
CSD number	2427252	2427233	2427245	2427232
Empirical formula	$(C_{16}H_{20}N_6NiO_6)_4 \cdot 5H_2O$	$C_{16}H_{20}N_6NiO_6 \cdot 2H_2O$	$[C_{20}H_{22}N_4NiO_6]_4 \cdot 9H_2O$	$[C_{20}H_{22}N_4NiO_6]_4 \cdot 9H_2O$
M/ g mol^{-1}	1894.43	487.12	513.66	513.66
Temperature /K	100(2)	100(2)	100(2)	100(2)
λ/ Å	1.54184	1.54184	1.54184	1.54184
Crystal System	Monoclinic	Monoclinic	Monoclinic	Monoclinic
Space group	P21	P21	C2	C2
Unit cell dimensions	a=20.2513(3) Å, b=10.15500(10) Å, c=24.3930(5) Å, α= 90° β=114.366(2)° γ= 90°	a=20.2855(2) Å, b=10.15990(10) Å, c=24.3927(3) Å, α= 90° β=114.5000(10)° γ= 90°	a=17.9584(3)) Å, b=7.29560(10) Å, c=17.8583(3) Å, α= 90° β=109.219(2)° γ= 90°	a=17.9600(3) Å, b=7.30610(10) Å, c=17.8397(3) Å, α= 90° β=109.147(2)° γ= 90°
Volume/ $Å^3$	4569.64(14)	4574.65(9)	2209.34(7)	2211.38(7)
Z	2	8	1	1
Abs. Coeff./mm^{-1}	1.655	1.694	1.770	1.768
F(000)	1972	2032	1074	1074
Crystal size/ mm^3	0.412x0.254x0.137	0.412x0.254x0.127	0.382x0.123x0.054	
Theta range/°	3.702 to 76.353	3.983 to 76.126	2.620 to 75.941	2.622 to 76.275
Index ranges	-25≤h≤25 -9≤k≤12 -30≤l≤30	-25≤h≤25 -8≤k≤12 -30≤l≤30	-22≤h≤22 -9≤k≤6 -21≤l≤22	-22≤h≤21 -7≤k≤9 -21≤l≤22
Reflections Collected	86687	86989	12301	20813
Independent Reflections	14297	14377	3392	4027
Flack param.	-0.04(3)	0.024(15)	-0.023(19)	-0.01(2)
R1	0.0608	0.0647	0.0255	0.0302
wR2	0.1513	0.1718	0.0675	0.0774
GOF on F^2	1.062	1.035	1.031	1.041

Supplemental Table 1 continued

Compound	**Ni(LβOHV^{Ox}4Im)$_2$**	**Ni(DβOHV^{Ox}4Im)$_2$**	**Ni(LβOHL^{Ox}4Im)$_2$**	**Ni(DβOHL^{Ox}4Im)$_2$**
CSD number	2427223	2453432	2427236	2427229
Empirical formula	$C_{18}H_{24}N_6NiO_6 \cdot H_2O$	$C_{18}H_{24}N_6NiO_6$	$C_{20}H_{28}N_6NiO_6$	$C_{20}H_{28}N_6NiO_6$
M/ g mol^{-1}	497.16	479.14	507.19	507.19
Temperature/ K	102(2)	102(2)	100(2)	100(2)
λ/ Å	1.54178	1.54184	1.54184	1.54184
Crystal System	Monoclinic	Monoclinic	Monoclinic	Monoclinic
Space group	C2	C2	C2	C2
Unit cell dimensions	a=22.12489(10) Å,<br>b=5.56335(4) Å,<br>c=8.69518(4) Å,<br>α= 90°<br>β=98.7630(4)°<br>γ= 90°	a=22.12885(14) Å,<br>b=5.55535(4) Å,<br>c=8.69173(6) Å,<br>α= 90°<br>β=98.8557(6)°<br>γ= 90°	a=24.2196(7) Å,<br>b=5.46280(10) Å,<br>c=8.7587(3) Å,<br>α= 90°<br>β=95.079(3)°<br>γ= 90°	a=24.1545(3) Å,<br>b=5.47860(10) Å,<br>c=8.77500(10) Å,<br>α= 90°<br>β=95.1700(10)°<br>γ= 90°
Volume/ Å^3	1057.783(10)	1055.767(12)	1154.29(6)	1156.50(3)
Z	2	2	2	2
Abs. Coeff./mm^{-1}	1.808	1.753	1.635	1.632
F(000)	520	500	532	532
Crystal size/ mm^3	0.439x0.165x0.116	0.249x0.155x0.104	0.26x0.03x0.015	0.316x0.093x0.025
Theta range/°	4.043to 76.227	4.044to 76.043	3.664 to 75.761	3.675 to 76.140
Index ranges	-27≤h≤27<br>-6≤k≤5<br>-10≤l≤10	-27≤h≤27<br>-6≤k≤6<br>-10≤l≤10	-30≤h≤28<br>-6≤k≤6<br>-10≤l≤11	-30≤h≤29<br>-6≤k≤6<br>-10≤l≤11
Reflections Collected	36228	19798	10575	19872
Independent Reflections	2062	2074	2091	2335
Flack param	-0.007(17)	-0.014(14)	-0.01(6)	0.06(4)
R1	0.0323	0.0350	0.0664	0.0398
wR2	0.0850	0.0929	0.1742	0.1101
GOF on F^2	1.051	1.099	1.073	1.084

Supplemental Table 1 continued

Compound	**Ni(LalloT^{Ald}5Me4Im)$_2$**	**Ni(LS^{Ald}5Me4Im)$_2$**	**Ni(DS^{Ald}5Me4Im)$_2$**
CSD number	2427241	2427243	2427230
Empirical formula	$(C_{18}H_{24}N_6NiO_6)_2 \cdot 3H_2O$	$C_{16}H_{20}N_6NiO_6 \cdot H_2O$	$C_{16}H_{20}N_6NiO_6 \cdot H_2O$
M/ g mol^{-1}	1012.33	469.10	469.10
Temperature/K	100(2)	100(2)	100(2)
λ/ Å	1.54178	1.54178	1.54178
Crystal System	Monoclinic	Trigonal	Trigonal
Space group	P21	P32	P32
Unit cell dimensions	a=9.70307(3) Å,<br>b=22.42884(8) Å,<br>c=10.42634(4) Å,<br>α= 90°<br>β=96.4243(3)°<br>γ= 90°	a=17.55462(5) Å,<br>b=17.55462(5) Å,<br>c=15.89838(6) Å,<br>α= 90°<br>β=90°<br>γ= 120°	a=17.55444(4) Å,<br>b=17.55444(4) Å,<br>c=15.90823(5) Å,<br>α= 90°<br>β=90°<br>γ= 120°
Volume/ Å^3	2254.819(14)	4242.93(3)	4245.47(2)
Z	2	9	9
Abs. Coeff./mm^{-1}	1.723	1.989	1.988
F(000)	1060	2196	2196
Crystal size/ mm^3	0.321x0.191x0.131	0.498x0.230x0.165	0.314x0.208x0.111
Theta range/°	3.942 to 76.267	2.779 to 76.299	2.778 to 76.269
Index ranges	$-12 \le h \le 12$<br>$-28 \le k \le 27$<br>$-13 \le l \le 13$	$-21 \le h \le 22$<br>$-21 \le k \le 22$<br>$-18 \le l \le 19$	$-22 \le h \le 22$<br>$-22 \le k \le 21$<br>$-18 \le l \le 19$
Reflections Collected	55342	115369	148804
Independent Reflections	9166	11562	11483
Flack param	-0.015(4)	-0.016(9)	-0.003(7)
R1	0.0234	0.0257	0.0241
wR2	0.0592	0.0686	0.0652
GOF on F^2	1.0028	1.058	1.053

Supplemental Table 2. Selected bond distances (Å) and angles (°) for the Ni(LβOHAA^{Ox}4Im)$_2$ and Ni(LT^{Ox}Py)$_2$ complexes. There is only one value for each entry of Ni(LβOHV^{Ox}4Im)$_2$ and Ni(LβOHL^{Ox}4Im)$_2$ as the asymmetric unit of each only contains one ligand. An atom marked with an ' indicates that it is an atom on the second of two identical ligands. For example O_{CA}-Ni-N_{Im} refers to two donor atoms on the same ligand and O_{CA}-Ni-N'_{Im} refers to donor atoms on different ligands.

Complex value	Ni(LT^{Ox}4Im)$_2$	Ni(LT^{Ox}Py)$_2$	Ni(LβOHV^{Ox}4Im)$_2$	Ni(LβOHL^{Ox}4Im)$_2$
Ni-O_{CA}	2.055(5)<br>2.054(6)	2.0294(17)<br>2.035(2)	2.063(2)	2.065(4)
Ni-N_{AA}	2.133(7)<br>2.100(7)	2.102(2)<br>2.0989(18)	2.130(3)	2.137(5)
Ni-$N_{Im/Py}$	2.036(7)<br>2.080(7)	2.0989(19)<br>2.0958(19)	2.046(2)	2.052(4)
$N_{Im/Py}$-Ni-$N'_{Im/Py}$	174.5(3)	173.42(18)	178.8(2)	179.9(4)
O_{CA}-Ni-$N'_{Im/Py}$	172.6(3)<br>172.6(3)	171.86(8)<br>170.07(7)	167.08(8)	165.76(15)
O_{CA}-Ni-$N_{Im/Py}$	92.8(3)<br>89.2(3)	92.53(7)<br>88.92(8)	93.97(10)	93.9(2)

Supplemental Table 3. Selected bond distances(Å) and angles(°) for the three five membered rings of the four Ni(LβOHAA^{Ox}Py/4Im)$_2$ complexes. Values for the D enantiomers are available from the CCDC and do not significantly differ from the L enantiomer

a) Ni(LT^{Ox}Py)$_2$ Pyridine: (Ni N_{Py} C_{Py} C_{ald} N_{AA}), Carboxylate: (Ni O_{CA} C_{CA} C_α N_{AA}) and Oxazolidine: (N_{AA} C_α C_β O_{Ox} C_{ald}) There are two values for each entry as there are two ligands in the asymmetric unit.

Pyridine	Value	Carboxylate	value	Oxazolidine	value
Ni- N_{Py}	2.0989(19)<br>2.0958(19)	Ni -O_{CA}	2.0294(17)<br>2.035(2)	N_{AA}-C_α	1.495(3)<br>1.494(4)
N_{Py}-C_{Py}	1.344(4)<br>1.347(3)	O_{CA}-C_{CA}	1.265(3)<br>1.260(3)	C_α-C_β	1.549(3)<br>1.553(3)
C_{Py}-C_{ald}	1.519(4)<br>1.520(3)	C_{CA}-C_α	1.523(4)<br>1.529(3)	C_β -O_{Ox}	1.442(3)<br>1.438(3)
C_{ald}-N_{AA}	1.470(3)<br>1.476(3)	C_α-N_{AA}	1.495(3)<br>1.496(4)	O_{Ox}-C_{ald}	1.410(3)<br>1.417(3)
N_{AA}-Ni	2.102(2)<br>2.0989(18)	N_{AA}-Ni	2.102(2)<br>2.0989(18)	C_{ald} -N_{AA}	1.470(3)<br>1.476(3)
Ni- N_{Py} C_{Py}	115.49(18)<br>115.24(16)	Ni -O_{CA}-C_{CA}	117.13(17)<br>116.38(17)	N_{AA}-C_α-C_β	105.0(2)<br>104.44(19)
N_{Py} -C_{Py}-C_{ald}	116.4(2)<br>115.9(2)	O_{CA}-C_{CA}-C_α	118.0(2)<br>118.8(2)	C_α -C_β -O_{Ox}	104.1(2)<br>104.4(2)
C_{Py}-C_{ald}-N_{AA}	111.2(2)<br>110.5(2)	C_{CA}-C_α-N_{AA}	112.9(2)<br>112.88(19)	C_β-O_{Ox}-C_{ald}	105.98(19)<br>105.28(18)
C_{ald}-N_{AA}-Ni	111.74(16)<br>111.24(14)	C_α-N_{AA}-Ni	108.88(16)<br>108.55(15)	O_{Ox} -C_{ald} -N_{AA}	106.70(19)<br>105.78(19)
N_{AA}-Ni-- N_{Py}	79.86(9)<br>80.12(7)	N_{AA}-Ni-O_{CA}	82.56(8)<br>82.79(9)	C_{ald}-N_{AA}-C_α	104.62(18)<br>104.42(18)

Supplemental Table 3 continued.

b) Ni(LT^{Ox}4Im)$_2$. Imidazole: (Ni N_{Im} C_{Im} C_{ald} N_{AA}), Carboxylate: (Ni O_{CA} C_{CA} C_{α} N_{AA}) and Oxazolidine: (N_{AA} C_{α} C_{β} O_{Ox} C_{ald}). There are two values for each entry as there are two ligands in the asymmetric unit.

Imidazole	Value	Carboxylate	value	Oxazolidine	value
Ni- N_{Im}	2.036(7)<br>2.080(7)	Ni -O_{CA}	2.055(5)<br>2.054(6)	N_{AA}-C_{α}	1.486(9)<br>1.501(10)
N_{Im}-C_{Im}	1.390(10)<br>1.378(12)	O_{CA}-C_{CA}	1.257(10)<br>1.282(10)	C_{α}-C_{β}	1.558(11)<br>1.551(11)
C_{Im}-C_{ald}	1.476(9)<br>1.485(11)	C_{CA}-C_{α}	1.527(10)<br>1.527(11)	C_{β} -O_{Ox}	1.449(8)<br>1.459(10)
C_{ald}-N_{AA}	1.476(9)<br>1.476(11	C_{α}-N_{AA}	1.486(9)<br>1.501(10)	O_{Ox}-C_{ald}	1.407(9)<br>1.417(10)
N_{AA}-Ni	2.133(7)<br>2.100(7)	N_{AA}-Ni	2.133(7)<br>2.100(7)	C_{ald} -N_{AA}	1.476(9)<br>1.476(11)
Ni- N_{Im} -C_{Im}	114.1(6)<br>113.4(5)	Ni -O_{CA}-C_{CA}	115.8(5)<br>114.4(5)	N_{AA}-C_{α}-C_{β}	105.4(5)<br>104.5(6)
N_{Im} -C_{Im}-C_{ald}	118.6(7)<br>119.1(7)	O_{CA}-C_{CA}-C_{α}	119.7(7)<br>119.6(7)	C_{α} -C_{β} -O_{Ox}	102.1(6)<br>103.6(6)
C_{Im}-C_{ald}-N_{AA}	109.9(6)<br>108.8(7)	C_{CA}-C_{α}-N_{AA}	112.3(6)<br>113.3(7)	C_{β}-O_{Ox}-C_{ald}	105.2(5)<br>105.9(6)
C_{ald}-N_{AA}-Ni	111.4(5)<br>112.7(5)	C_{α}-N_{AA}-Ni	109.4(4)<br>108.1(5)	O_{Ox} -C_{ald} -N_{AA}	106.7(6)<br>106.2(7)
N_{AA}-Ni—N_{Im}	81.3(3)<br>80.1(3)	N_{AA}-Ni-O_{CA}	81.3(2)<br>83.8(2)	C_{ald}-N_{AA}-C_{α}	104.3(6)<br>104.4(6)

Supplemental Table 3 continued.

c) Ni(LβOHV^{Ox}4Im)$_2$. Imidazole: (Ni N_{Im} C_{Im} C_{ald} N_{AA}), Carboxylate: (Ni O_{CA} C_{CA} C_α N_{AA}) and Oxazolidine: (N_{AA} C_α C_β O_{Ox} C_{ald}). There is only one value for each entry as there is only a single ligand in the asymmetric unit.

Imidazole	Value	carboxylate	value	oxazolidine	value
Ni-N_{Im}	2.046(2)	Ni -O_{CA}	2.063(2)	N_{AA}-C_α	1.488(4)
N_{Im} -C_{Im}	1.373(4)	O_{CA}-C_{CA}	1.265(4)	C_α-C_β	1.570(4)
C_{Im} -C_{ald}	1.505(4)	C_{CA}-C_α	1.528(4)	C_β -O_{Ox}	1.442(4)
C_{ald}-N_{AA}	1.487(4)	C_α-N_{AA}	1.488(4)	O_{Ox}-C_{ald}	1.413(3)
N_{AA}-Ni	2.130(3)	N_{AA}-Ni	2.130(3)	C_{ald} -N_{AA}	1.487(4)
Ni- N_{Im} -C_{Im}	115.9(2)	Ni -O_{CA}-C_{CA}	114.8(2)	N_{AA}-C_α-C_β	105.7(2)
N_{Im} -C_{Im}-C_{ald}	118.6(2)	O_{CA}- C_{CA}-C_α	118/3(3)	C_α -C_β -O_{Ox}	102.9(2)
C_{Im}-C_{ald}-N_{AA}	109.9(2)	C_{CA}-C_α-N_{AA}	113.1(2)	C_β-O_{Ox}-C_{ald}	108.5(2)
C_{ald}-N_{AA}-Ni	112.79(18)	C_α-N_{AA}-Ni	108.66(19)	O_{Ox} -C_{ald} -N_{AA}	106.6(2)
N_{AA}-Ni—N_{im}	80.28(10)	N_{AA}-Ni-O_{CA}	81.58(9)	C_{ald}-N_{AA}-C_α	105.7(2)

d) Ni(LβOHL^{Ox}4Im)$_2$. Imidazole: (Ni N_{Im} C_{Im} C_{ald} N_{AA}), Carboxylate: (Ni O_{CA} C_{CA} C_α N_{AA}) and Oxazolidine: (N_{AA} C_α C_β O_{Ox} C_{ald}). There is only one value for each entry as there is only a single ligand in the asymmetric unit.

Imidazole	Value	carboxylate	value	oxazolidine	value
Ni-N_{Im}	2.052(4)	Ni -O_{CA}	2.065(4)	N_{AA}-C_α	1.474(7)
N_{Im} -C_{Im}	1.369(8)	O_{CA}-C_{CA}	1.278(7)	C_α-C_β	1.564(7)
C_{Im} -C_{ald}	1.505(8)	C_{CA}-C_α	1.516(8)	C_β -O_{Ox}	1.429(7)
C_{ald}-N_{AA}	1.498(7)	C_α-N_{AA}	1.474(7)	O_{Ox}-C_{ald}	1.420(7)
N_{AA}-Ni	2.137(5)	N_{AA}-Ni	2.137(5)	C_{ald} -N_{AA}	1.498(7)
Ni- N_{Im} -C_{Im}	116.0(4)	Ni -O_{CA}-C_{CA}	113.6(4)	N_{AA}-C_α-C_β	104.2(4)
N_{Im} -C_{Im}-C_{ald}	118.8(5)	O_{CA}- C_{CA}-C_α	117.7(5)	C_α -C_β -O_{Ox}	103.3(4)
C_{Im}-C_{ald}-N_{AA}	109.2(5)	C_{CA}-C_α-N_{AA}	113.6(5)	C_β-O_{Ox}-C_{ald}	105.7(4)
C_{ald}-N_{AA}-Ni	112.9(4)	C_α-N_{AA}-Ni	108.4(4)	O_{Ox} -C_{ald} -N_{AA}	105.9(4)
N_{AA}-Ni—N_{im}	80.2(2)	N_{AA}-Ni-O_{CA}	81.15(17)	C_{ald}-N_{AA}-C_α	106.0(4)

Supplemental Table 4. Crystallographic data for **supplemental structures of 2Me4Im condensed with select beta hydroxy amino acids**

Compound	**Ni(LT^{Ald}2Me4Im)$_2$**	**Ni(LalloT^{Ald}2Me4Im)$_2$**	**Ni(LβOHV^{Ald}2Me4Im)$_2$**	**Ni(LβOHV^{Ox}2Me4Im)(LβOHV)**
CSD number	2427219	2427237	242716	2427218
Empirical formula	$C_{18}H_{24}N_6O_6Ni \cdot 4H_2O$	$C_{18}H_{24}N_6O_6Ni \cdot 5H_2O$	$C_{20}H_{28}N_6O_6Ni \cdot 5H_2O$	$C_{15}H_{24}N_4O_6Ni \cdot 3H_2O$
M/ g mol^{-1}	551.20	569.22	597.27	469.14
Temperature/K	293(2)	100(2)	100(2)	100(2)
λ/ Å	1.54184	1.54184	1.54184	1.54184
Crystal System	Orthorhombic	Orthorhombic	Orthorhombic	Monoclinic
Space group	P $2_12_12_1$	P $2_12_12_1$	P $2_12_12_1$	C 2
Unit cell dimensions	a=16.0006(3) Å,<br>b=15.2949(3) Å,<br>c=10.2243(2) Å,<br>α= 90°<br>β= 90°<br>γ= 90°	a=7.98550(3) Å,<br>b=15.57929(6) Å,<br>c=20.58119(11)Å,<br>α= 90°<br>β=90°<br>γ= 90°	a=8.6074(2) Å,<br>b=16.4991(4) Å,<br>c=19.5926(3) Å,<br>α= 90°<br>β=90°<br>γ= 90°	a=20.6854(6) Å,<br>b=5.56600(10) Å<br>c=18.0822(3) Å,<br>α= 90°<br>β=112.662(7)°<br>γ= 90°
Volume/ Å^3	2502.17(8)	2560.47(2)	2782.43(10)	2018.05(8)
Z	4	4	4	4
Abs. Coeff./mm^{-1}	1.675	1.684	1.576	1.894
F(000)	1160	1200	1264	992
Crystal size/ mm^3	0.187x0.092x0.067	0.299x0.172x0.102	0.22x0.15x0.10	0.484x0.040x0.023
Theta range/°	3.998 to76.244	3.558 to 76.186	3.502 to 76.406	2.521 to 76.169
Index ranges	$-18 \leq h \leq 20$<br>$-19 \leq k \leq 19$<br>$-12 \leq l \leq 12$	$-10 \leq h \leq 9$<br>$-19 \leq k \leq 17$<br>$-25 \leq l \leq 25$	$-10 \leq h \leq 10$<br>$-17 \leq k \leq 20$<br>$-24 \leq l \leq 24$	$-26 \leq h \leq 25$<br>$-6 \leq k \leq 5$<br>$-22 \leq l \leq 22$
Reflections Collected	50175	49044	67778	23033
Independent Reflections	5104	5269	5751	3822
Flack parameter	-0.032(13)	-0.0148	-0.027(12)	-0.032
R1	0.0772	0.0244	0.0415	0.0367
wR2	0.2086	0.0630	0.1067	0.0973
GOF on F^2	1.097	1.036	1.045	1.052

Supplemental Table 5 Summary of %yield, EA and ESI MS values for one enantiomer of the prepared complexes

Complex	%Yield	Theoretical CHN (%)	Experimental CHN (%)	ESI (m/e)
Ni(LT^{Ox}4Im)$_2$· 4.5H_2O<br>$C_{16}H_{20}N_6O_6Ni$ (450)	50.4%	36.11<br>5.49<br>15.79	36.02<br>:5.35<br>15.59	$[M+H]^+$ 451<br>$[M+Na]^+$ 473
Ni(LT^{Ox}Py)$_2$·2.5H_2O<br>$C_{20}H_{22}N4O_6Ni$ (472)	23.9%	46.36<br>5.25<br>10.81	46.19<br>5.02<br>10.41	$[M+H]^+$ 473<br>$[M+Na]^+$ 495<br>$[M+K]^+$ 511
Ni(LβOHV^{Ox}4Im)$_2$·0.5H_2O<br>$C_{18}H_{24}N_6O_6Ni$ (478)	34.4%	44.29<br>5.16<br>17.22	44.65<br>5.15<br>17.11	$[M+H]^+$ 479<br>$[M+Na]^+$ 501<br>$[M+K]^+$ 517
Ni(DβOHL^{Ox}4Im)$_2$·0.5H_2O<br>$C_{20}H_{28}N_6O_6Ni$ (506)	46.1%	46.54<br>5.66<br>16.28	46.71<br>5.51<br>16.10	$[M+Na]^+$ 529
Ni(LS^{Ald}5Me4Im)$_2$·1.5H_2O<br>$C_{16}H_{20}N_6O_6Ni$ (450)	58.1%	40.2<br>4.85<br>17.58	40.32<br>4.60<br>17.29	$[M+Na]^+$ 473
Ni(LalloT^{Ald}5Me4Im)$_2$·2H_2O<br>$C_{18}H_{24}N_6O_6Ni$ (478)	46.2%	41.97<br>5.48<br>16.31	41.93<br>5.26<br>16.20	$[M+Na]^+$ 501

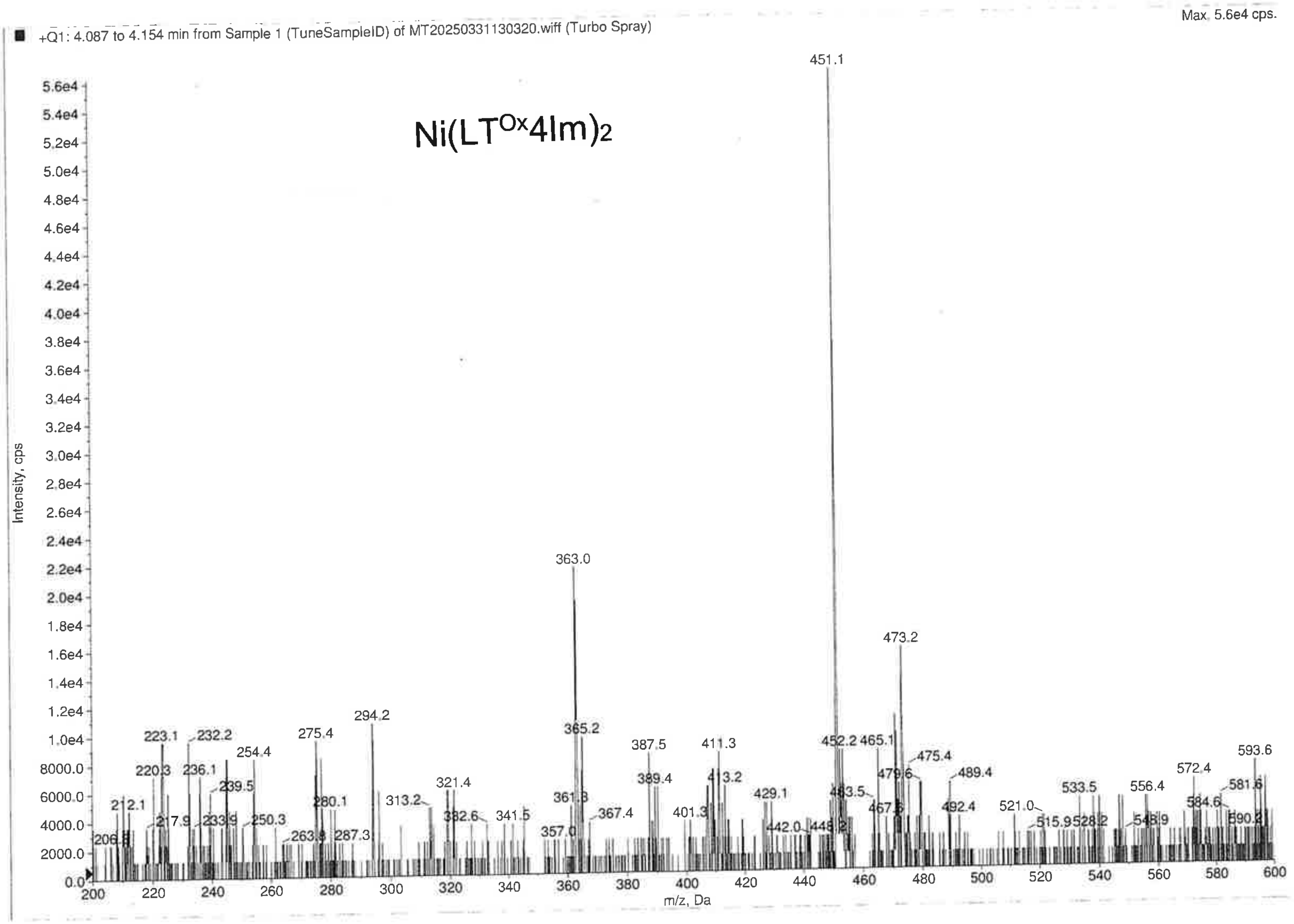

+Q1: 4.087 to 4.154 min from Sample 1 (TuneSampleID) of MT20250331130320.wiff (Turbo Spray)
Max. 5.6e4 cps.
Ni(LT^Ox4Im)2
Intensity, cps
m/z, Da
451.1
363.0
473.2
294.2
365.2
223.1
232.2
275.4
254.4
387.5
411.3
452.2
465.1
475.4
593.6
572.4
489.4
389.4
413.2

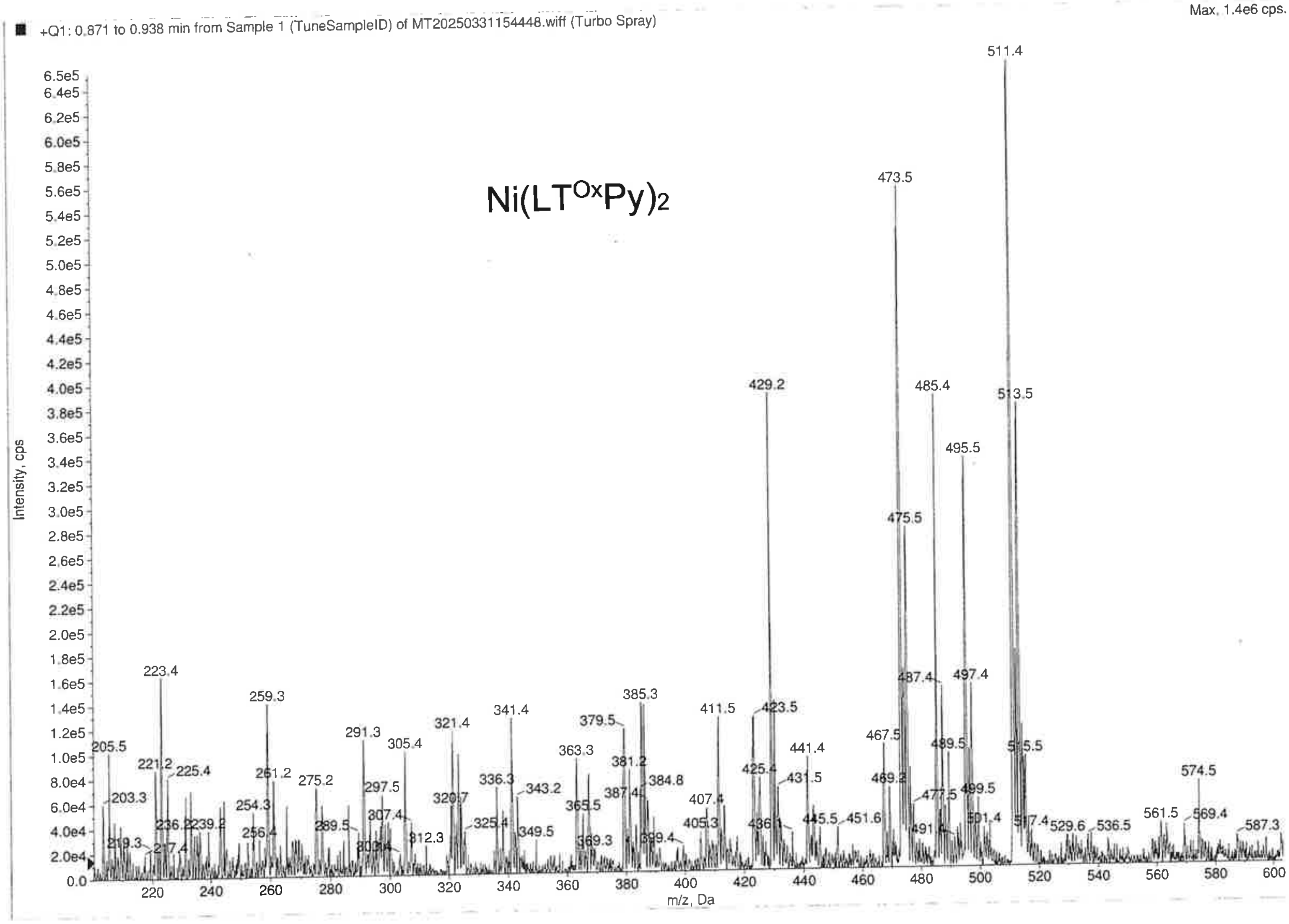
+Q1: 0.871 to 0.938 min from Sample 1 (TuneSampleID) of MT20250331154448.wiff (Turbo Spray)
Max. 1.4e6 cps.
Ni(LT^OxPy)2
Intensity, cps
m/z, Da
511.4
473.5
429.2
485.4
513.5
495.5
475.5
223.4
259.3
385.3
411.5
423.5
487.4
497.4
341.4
321.4
291.3
379.5
305.4
363.3
441.4
467.5
489.5
515.5
574.5

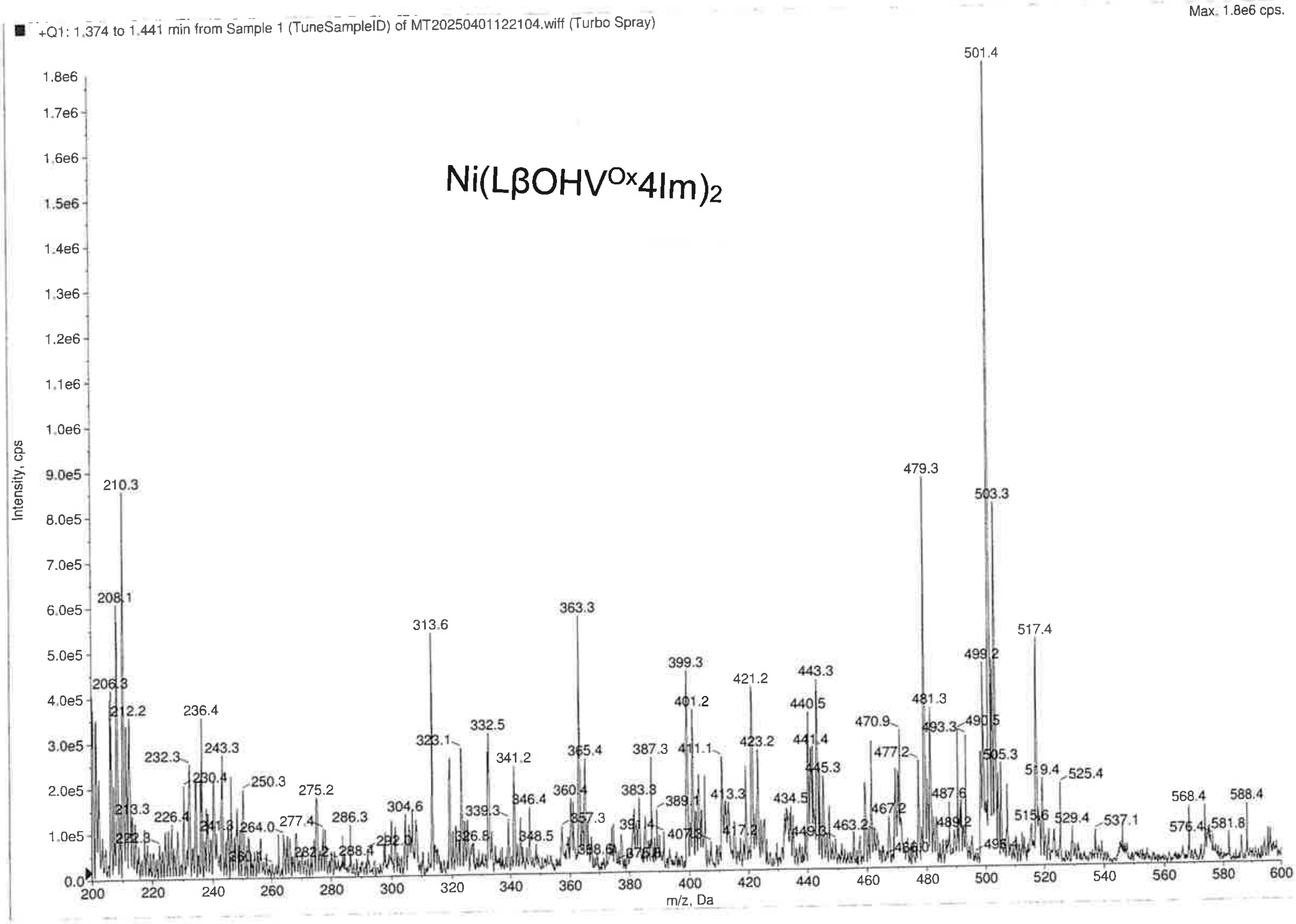
+Q1: 1.374 to 1.441 min from Sample 1 (TuneSampleID) of MT20250401122104.wiff (Turbo Spray)
Max. 1.8e6 cps.
Ni(LβOHVOx4Im)2
Intensity, cps
m/z, Da
501.4
479.3
503.3
210.3
208.1
363.3
313.6
517.4
499.2
443.3
421.2
399.3
401.2
481.3
236.4

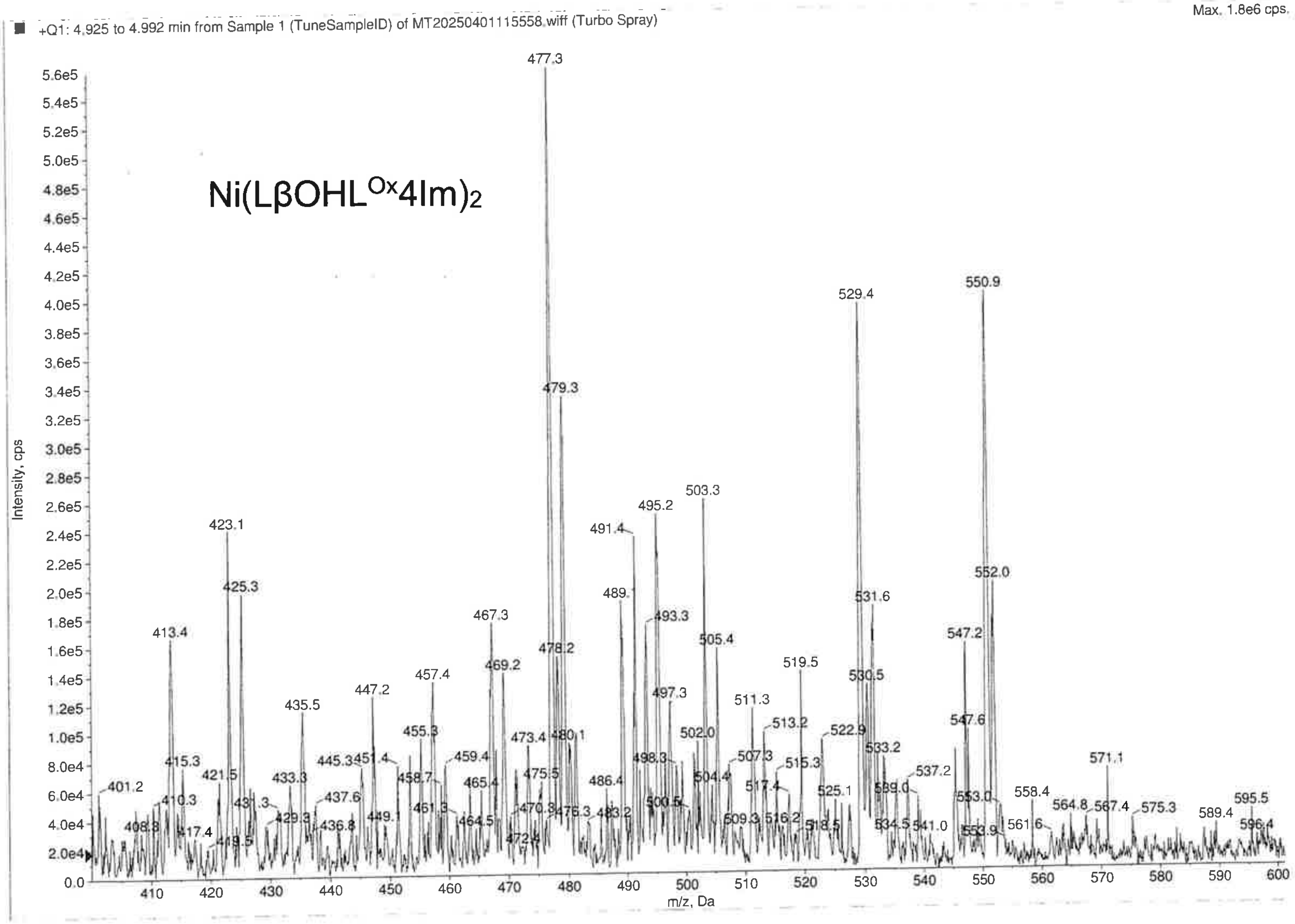
+Q1: 4.925 to 4.992 min from Sample 1 (TuneSampleID) of MT20250401115558.wiff (Turbo Spray)
Max. 1.8e6 cps.
Ni(LβOHL^Ox4Im)2
Intensity, cps
m/z, Da
477.3
479.3
529.4
550.9
552.0
531.6
503.3
495.2
491.4
489.1
493.3
505.4
423.1
425.3
413.4
467.3
469.2
478.2
547.2
519.5
530.5
457.4
447.2
435.5
497.3
511.3
513.2
522.9
547.6
571.1

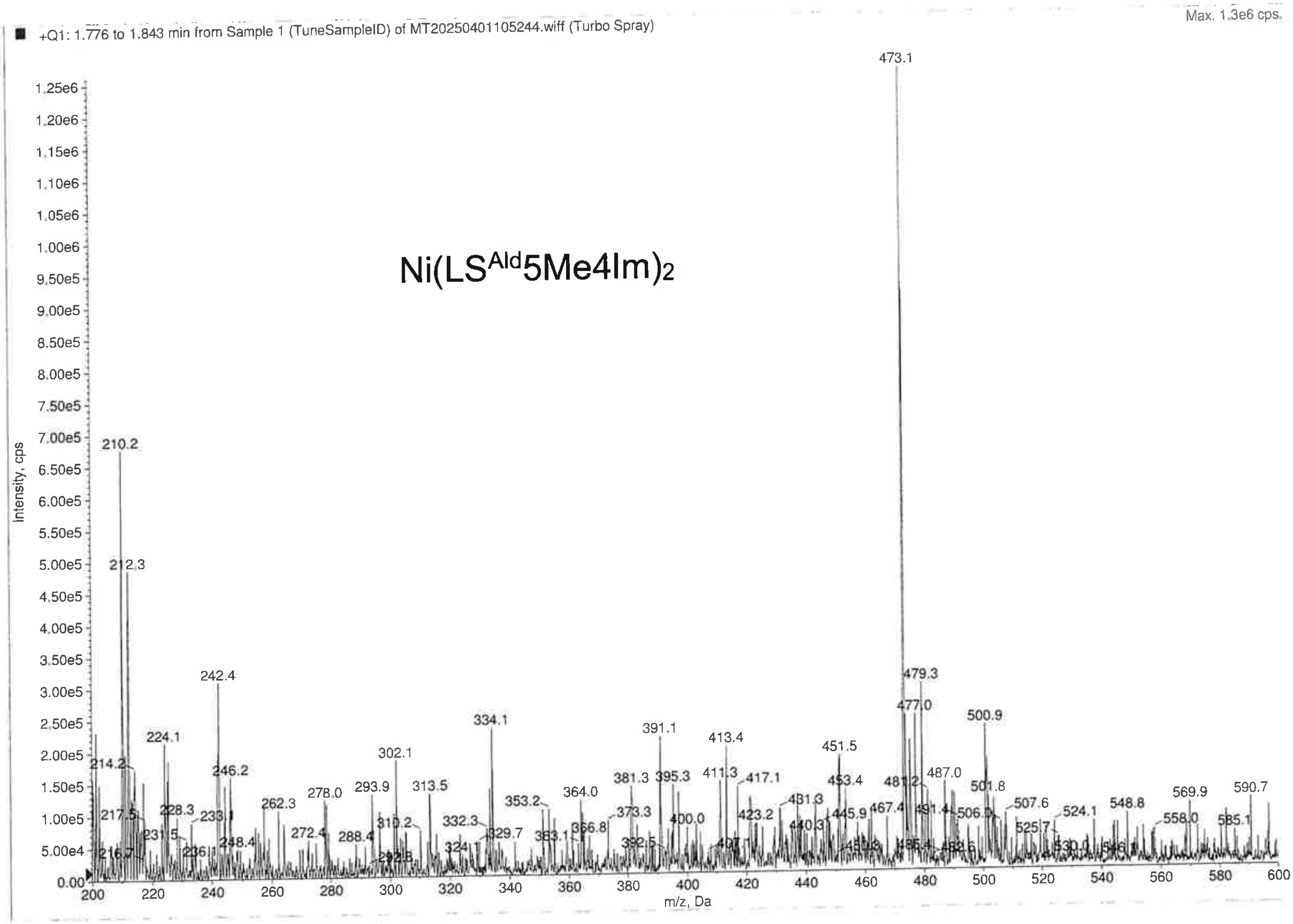
+Q1: 1.776 to 1.843 min from Sample 1 (TuneSampleID) of MT20250401105244.wiff (Turbo Spray)
Max. 1.3e6 cps.
Ni(LS^Ald5Me4Im)2
Intensity, cps
m/z, Da
473.1
210.2
212.3
242.4
479.3
477.0
500.9
334.1
391.1
413.4
451.5
302.1
224.1
487.0
590.7
569.9
548.8
524.1

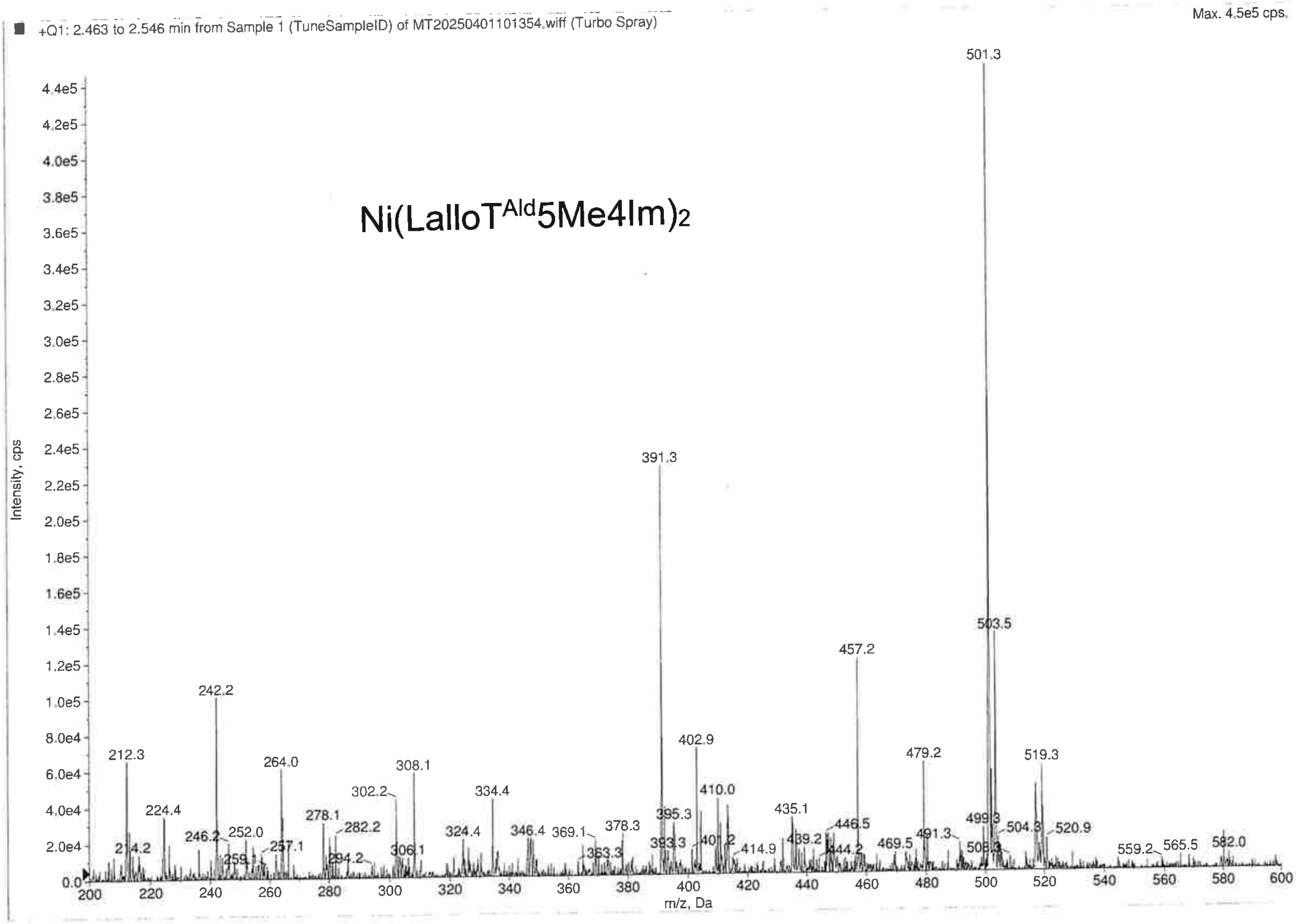

+Q1: 2.463 to 2.546 min from Sample 1 (TuneSampleID) of MT20250401101354.wiff (Turbo Spray)
Max. 4.5e5 cps.
Ni(LalloT^Ald5Me4Im)2
Intensity, cps
m/z, Da
501.3
391.3
503.5
457.2
242.2
402.9
479.2
519.3
212.3
264.0
308.1
410.0
302.2
334.4
435.1
224.4
395.3
446.5
278.1
282.2
378.3
499.3
504.3
520.9
252.0
246.2
324.4
346.4
369.1
491.3
469.5
257.1
214.2
306.1
393.3
414.9
439.2
444.2
363.3
259.1
294.2
508.3
559.2
565.5
582.0

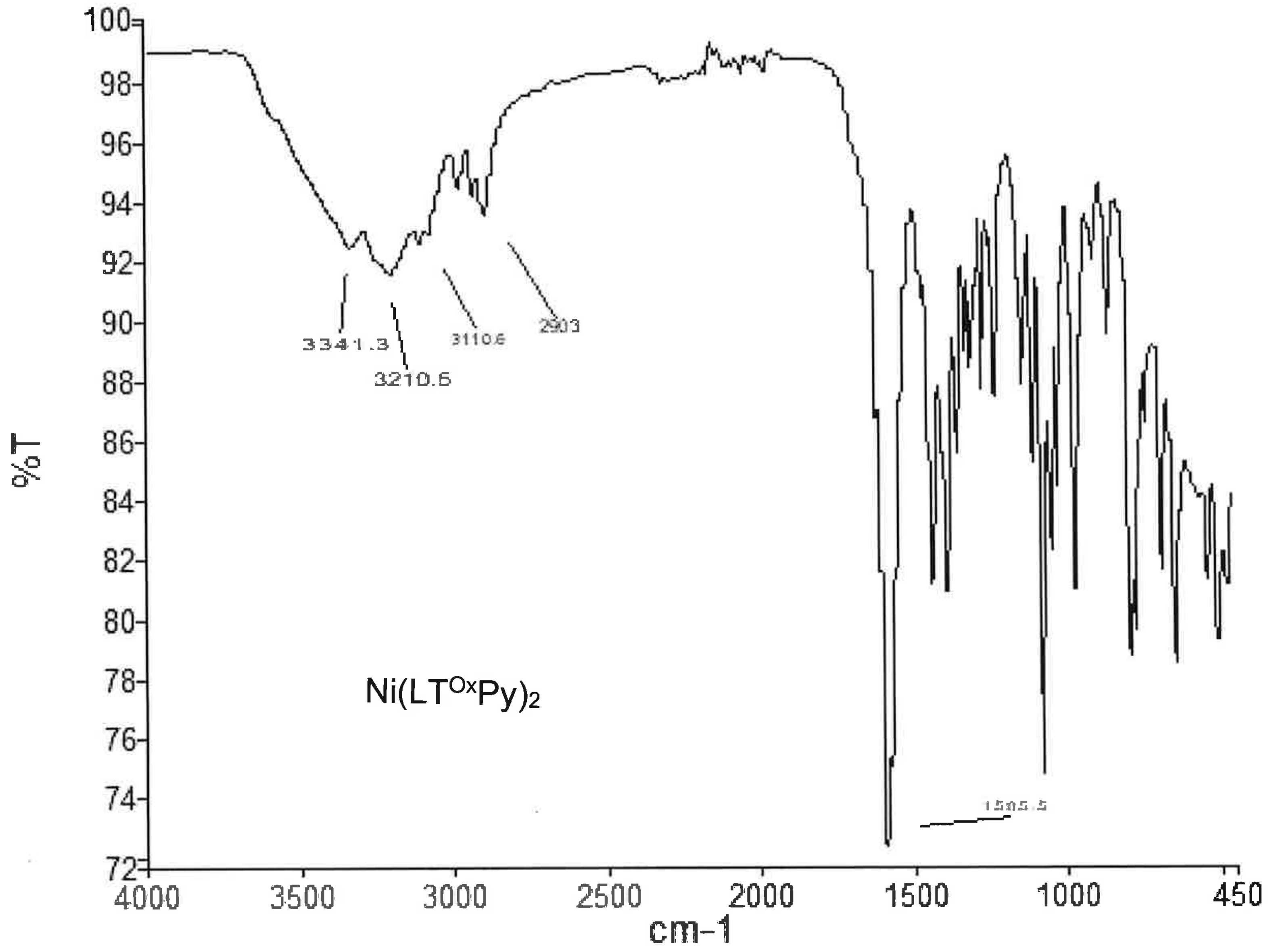
100
98
96
94
92
90
88
86
84
82
80
78
76
74
72
%T
3341.3
3210.6
3110.6
2903
1595.5
Ni(LT^Ox Py)2
4000
3500
3000
2500
2000
1500
1000
450
cm-1

J70 pyLT

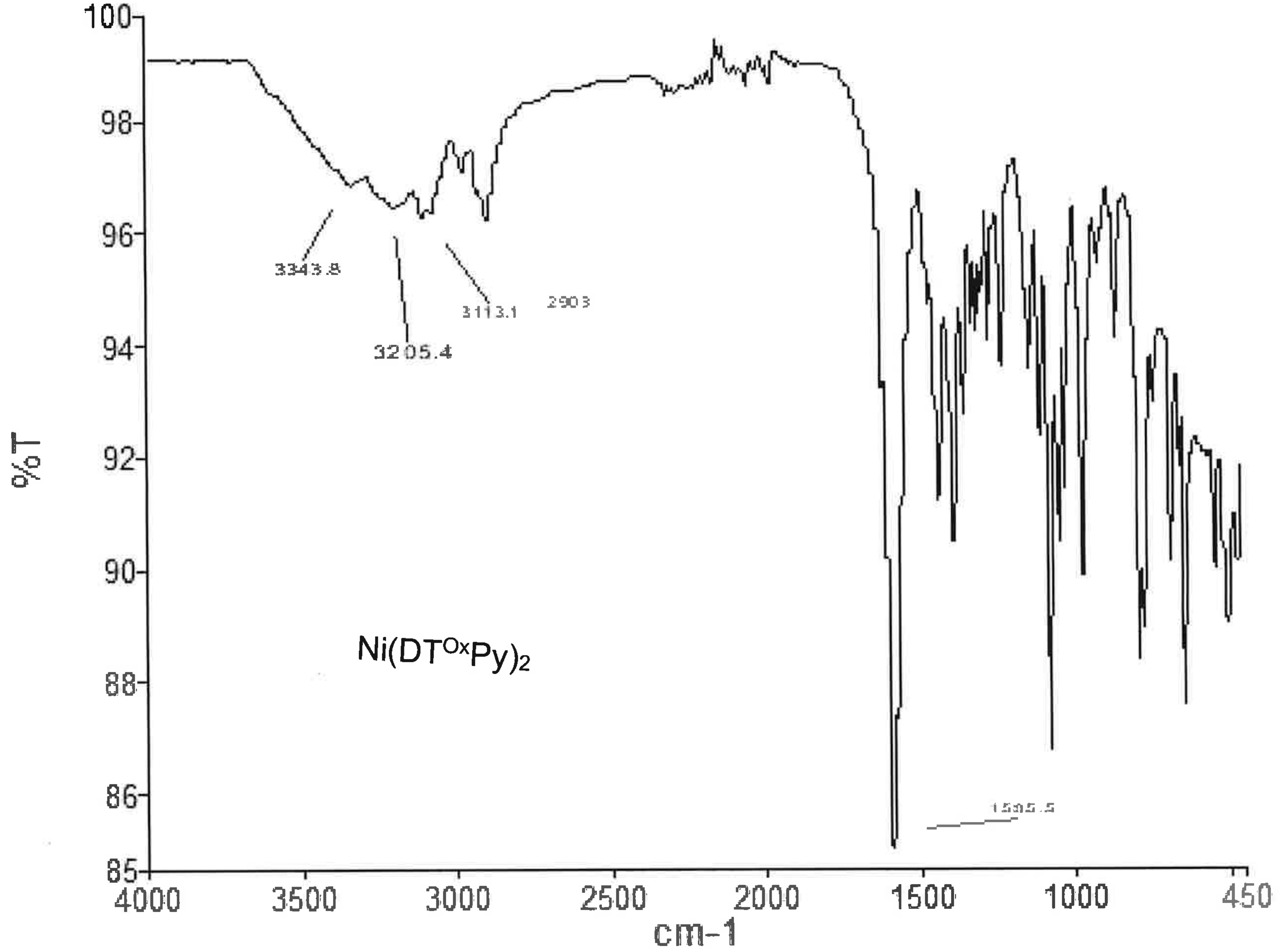
%T
100
98
96
94
92
90
88
86
85
4000
3500
3000
2500
2000
1500
1000
450
cm-1
3343.8
3205.4
3113.1
2903
1595.5
Ni(DT^Ox Py)2

K1 pyCHO DT

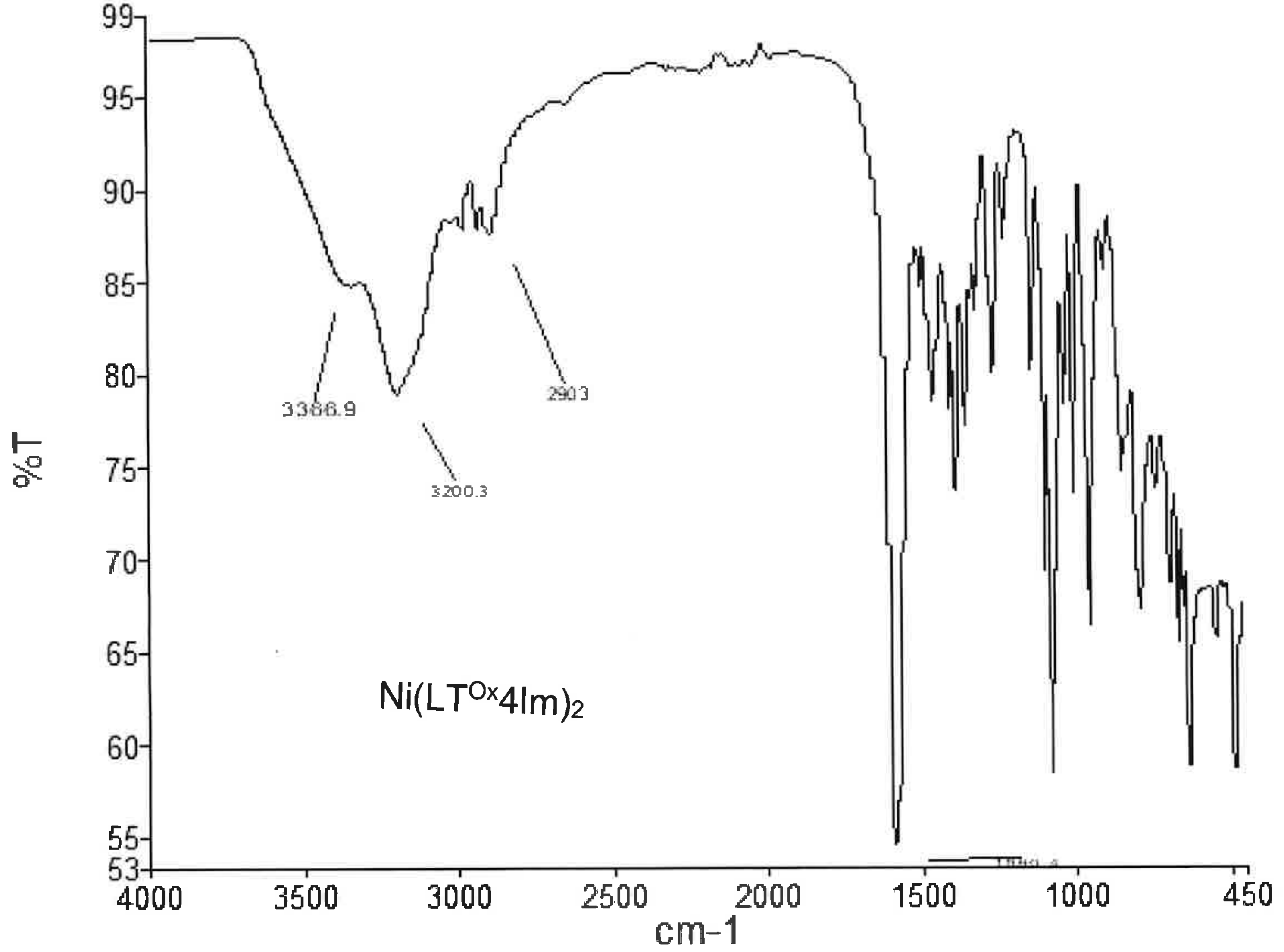
99
95
90
85
80
75
70
65
60
55
53
%T
4000
3500
3000
2500
2000
1500
1000
450
cm-1
3386.9
3200.3
2903
Ni(LT^Ox4Im)2

J58 4ImLT

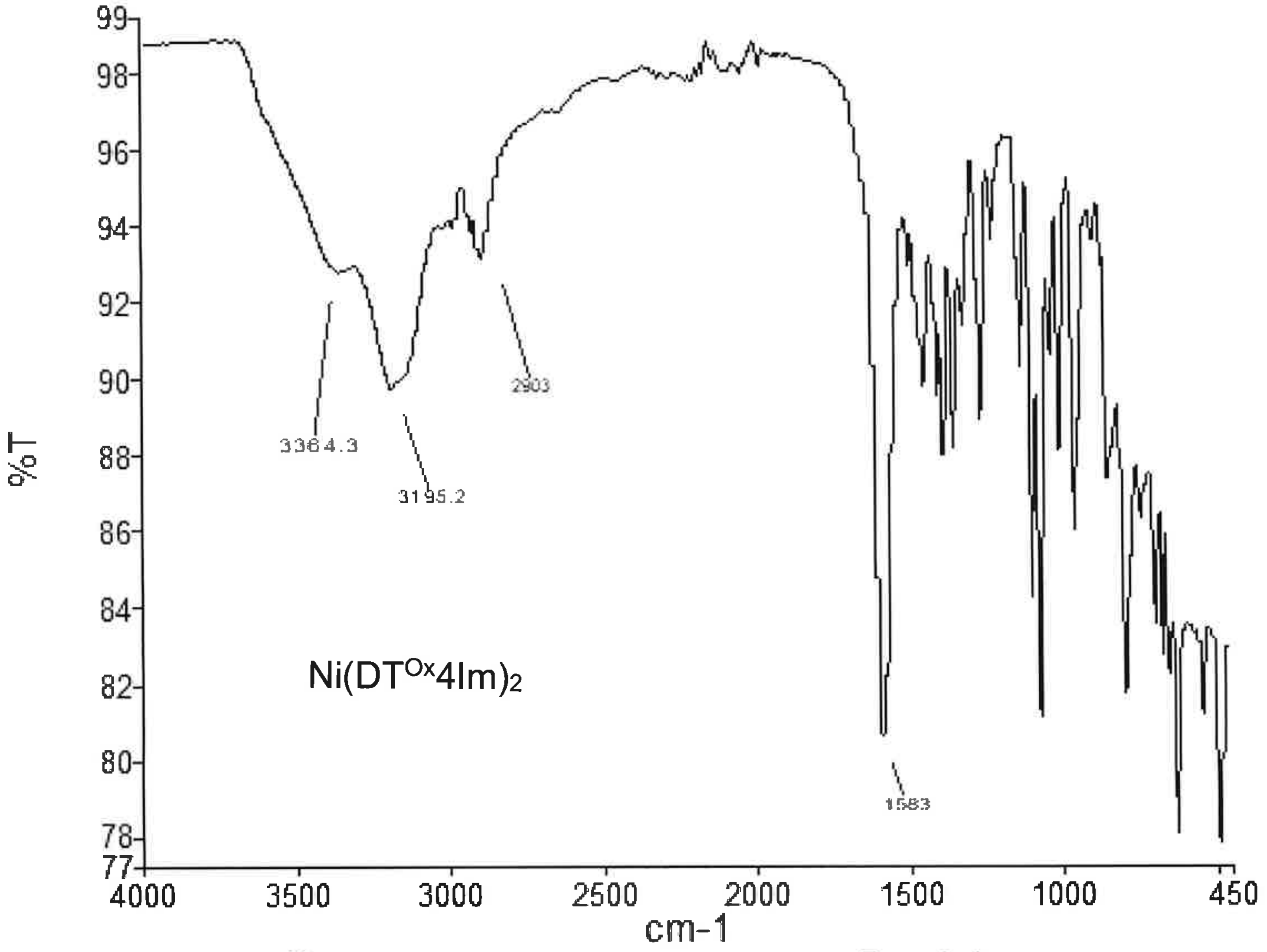
99
98
96
94
92
90
88
86
84
82
80
78
77
%T
3364.3
3195.2
2903
1583
Ni(DT^Ox 4Im)2
4000
3500
3000
2500
2000
1500
1000
450
cm-1

J58 4ImDT

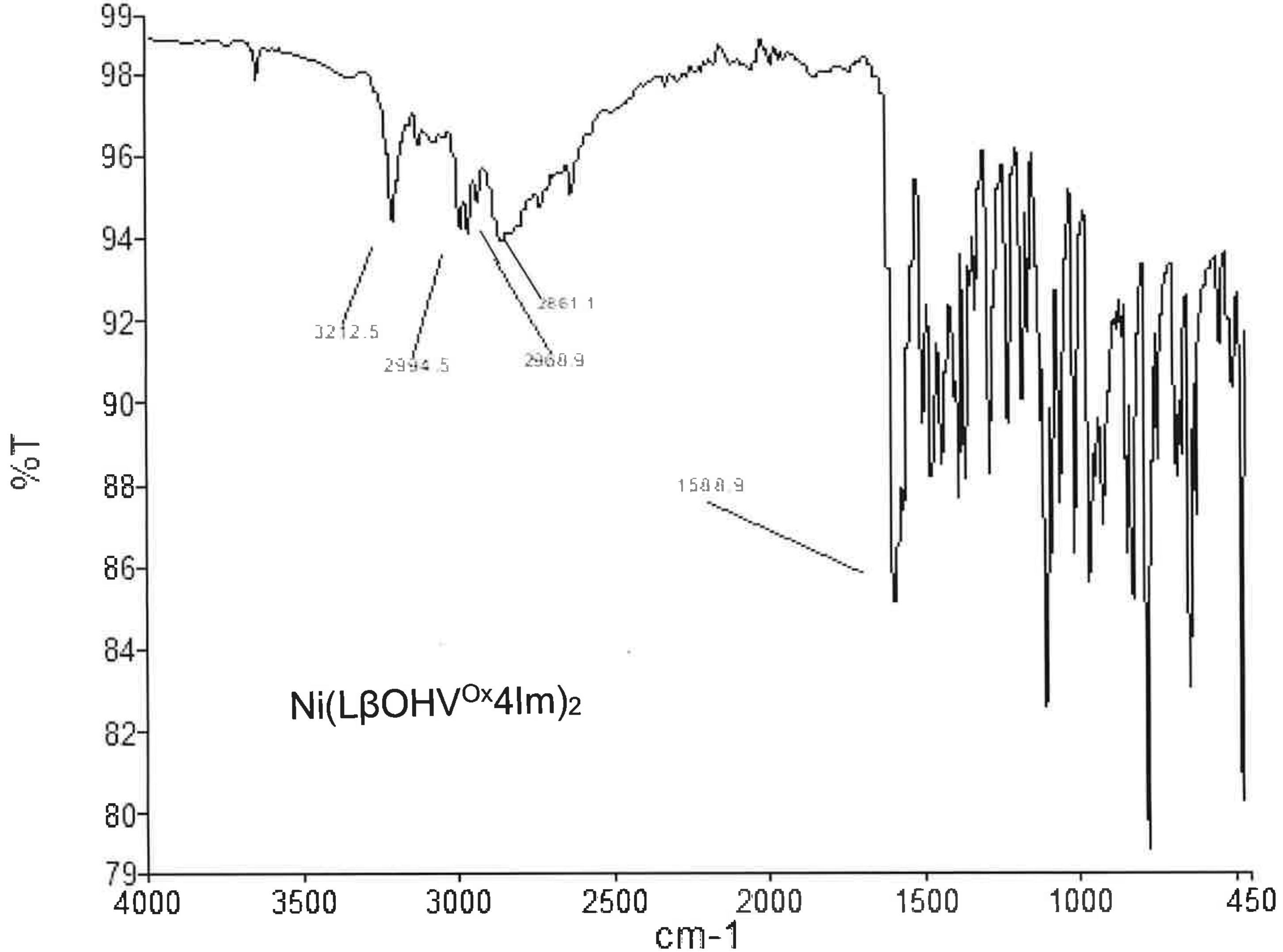

Ctb50-1 LβOHV+4Im

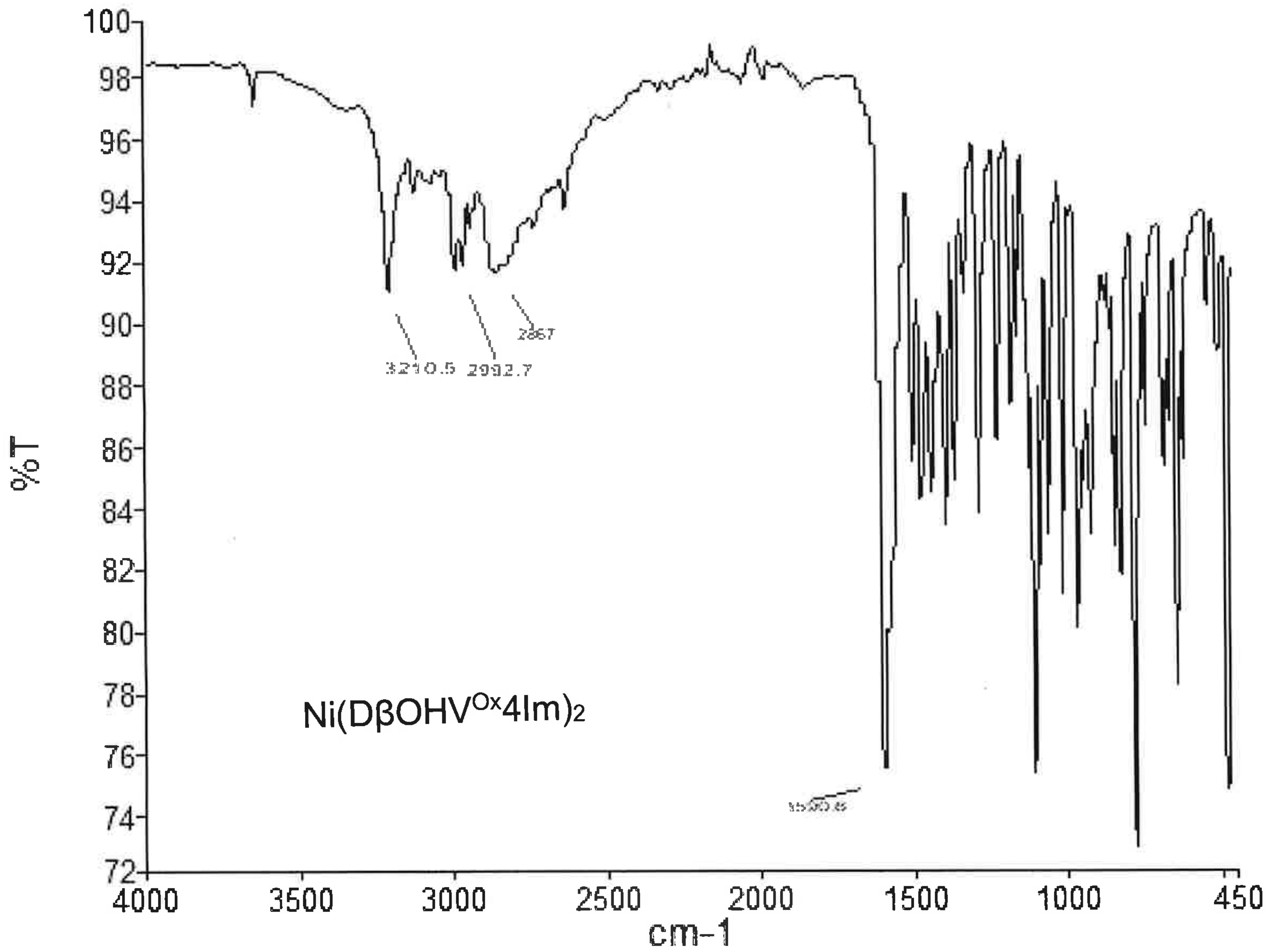

K74 DβOHV+4Im

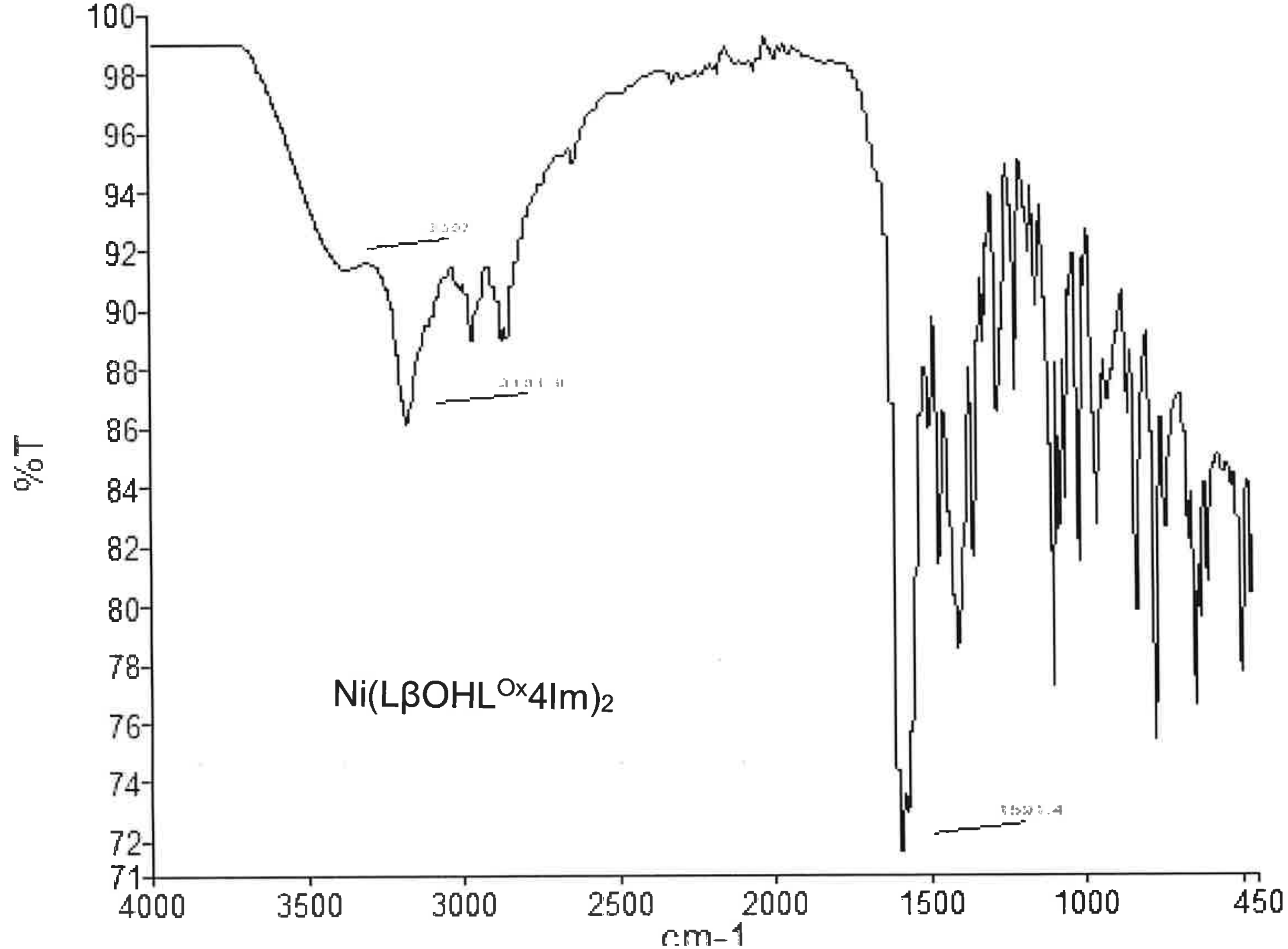

K57-1 isoPrLT+4Im

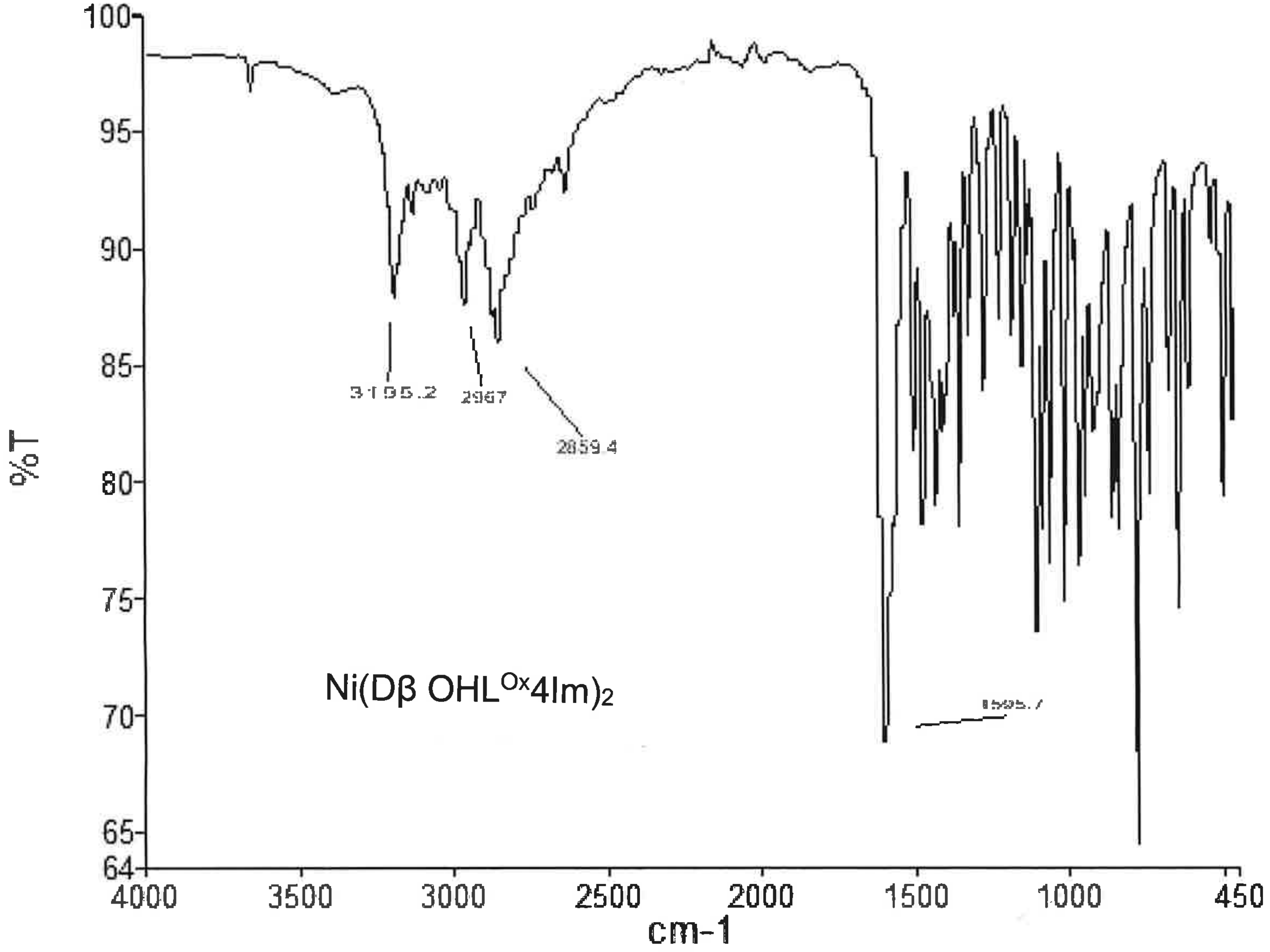

K30 isoPrDT+4Im

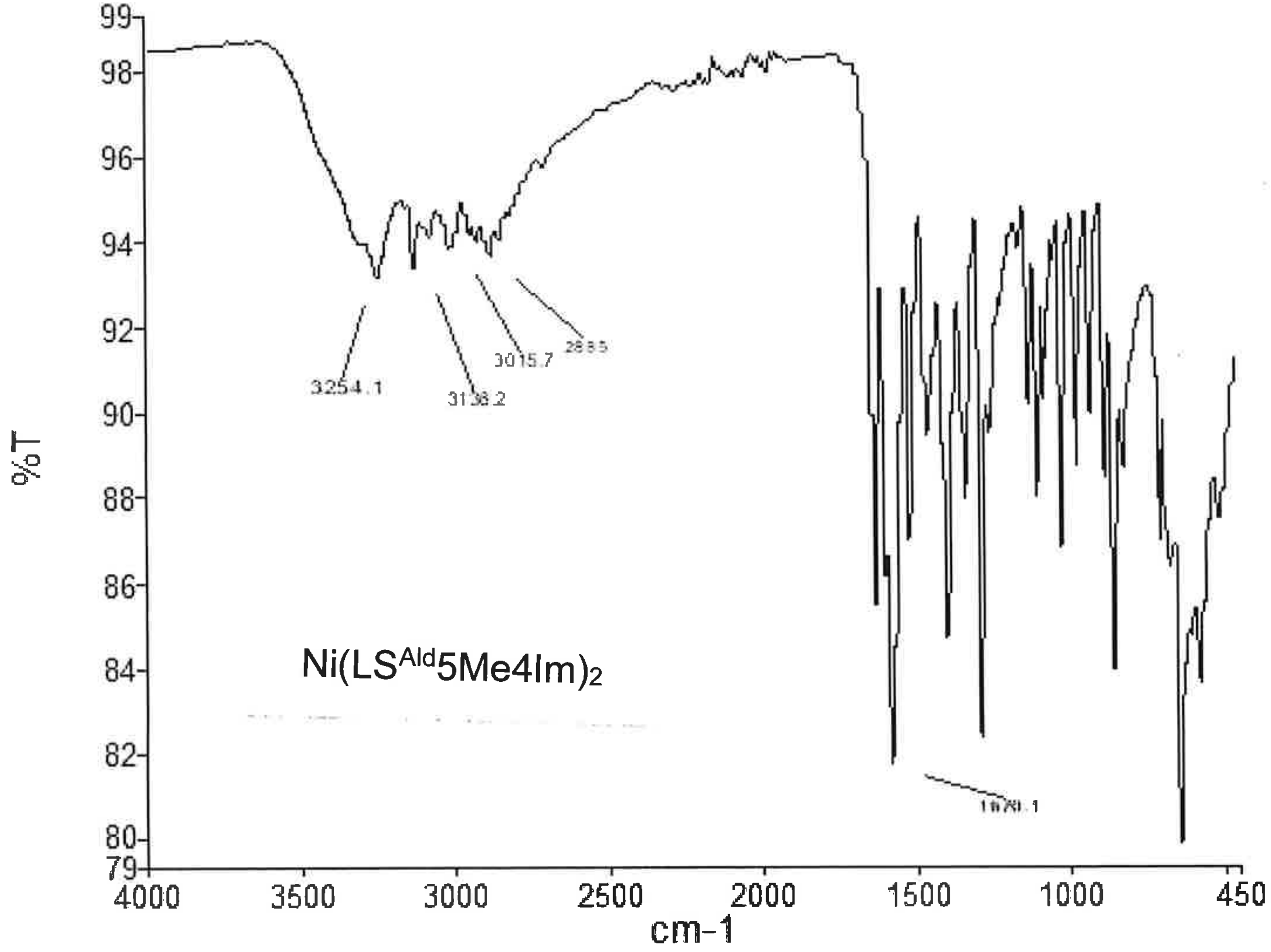

J68 5Me4ImLS

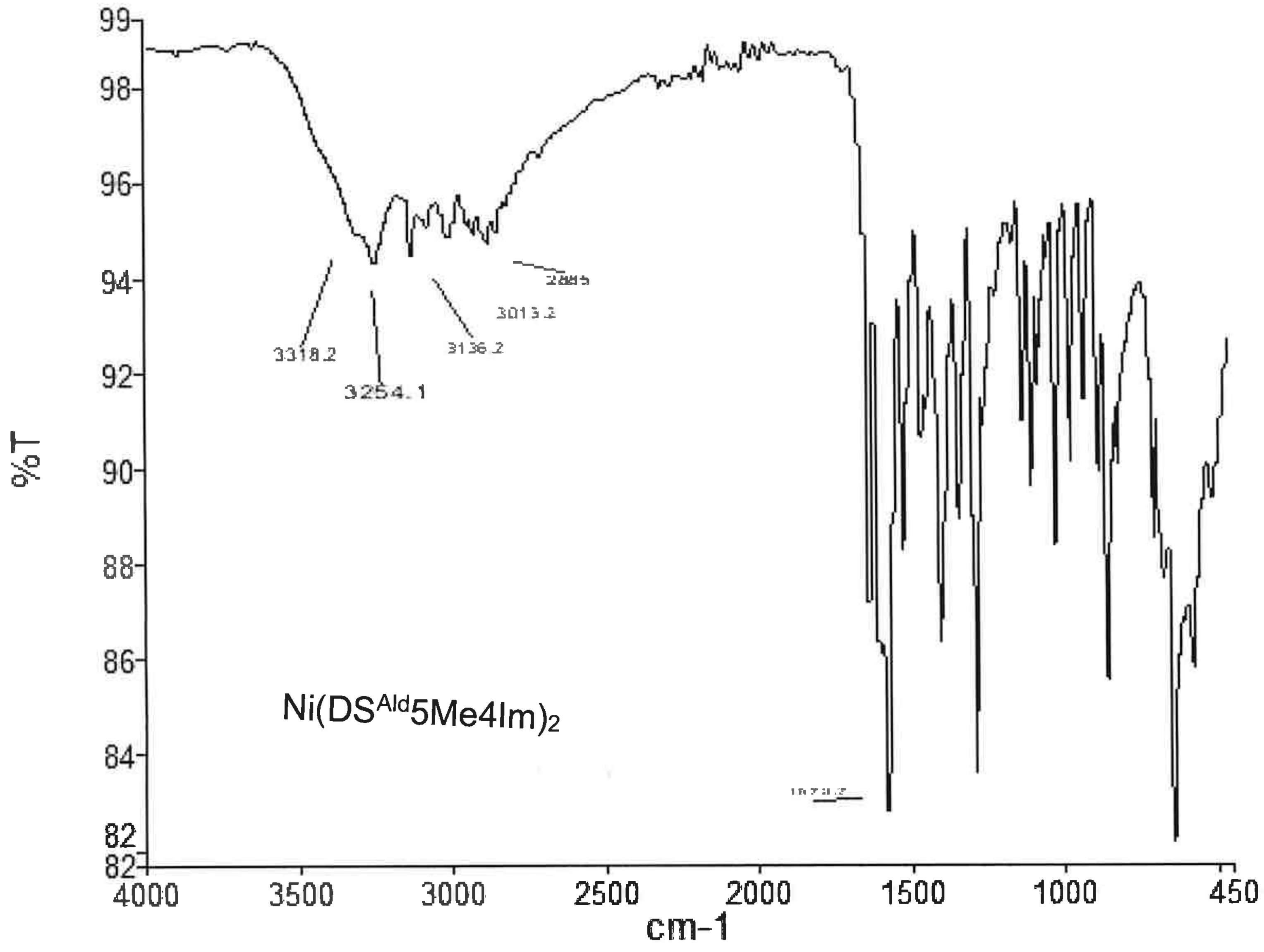

J68 5Me4ImDS

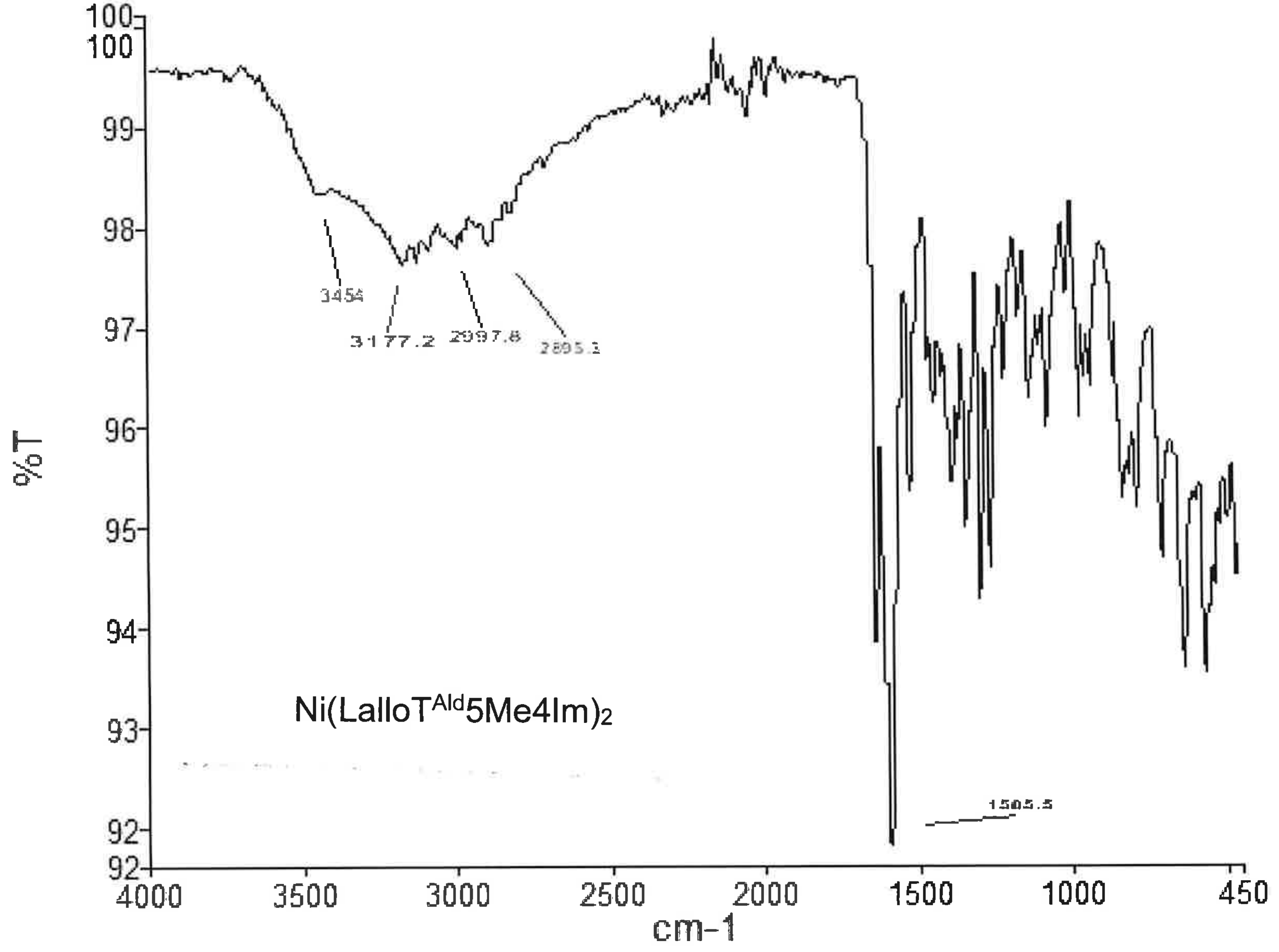

K19 LAT+5Me4Im
